# Internet‐of‐medical‐things integrated point‐of‐care biosensing devices for infectious diseases: Toward better preparedness for futuristic pandemics

**DOI:** 10.1002/btm2.10481

**Published:** 2023-01-03

**Authors:** Arpana Parihar, Shalu Yadav, Mohd Abubakar Sadique, Pushpesh Ranjan, Neeraj Kumar, Ayushi Singhal, Vedika Khare, Raju Khan, Sathish Natarajan, Avanish K. Srivastava

**Affiliations:** ^1^ Industrial Waste Utilization, Nano and Biomaterials, CSIR‐Advanced Materials and Processes Research Institute (AMPRI) Bhopal Madhya Pradesh India; ^2^ Academy of Scientific and Innovative Research (AcSIR) Ghaziabad India; ^3^ School of Nanotechnology, UTD, RGPV Campus Bhopal Madhya Pradesh India

**Keywords:** electrochemical, infectious disease, internet‐of‐medical‐things, microbial sensors, microfluidic

## Abstract

Microbial pathogens have threatened the world due to their pathogenicity and ability to spread in communities. The conventional laboratory‐based diagnostics of microbes such as bacteria and viruses need bulky expensive experimental instruments and skilled personnel which limits their usage in resource‐limited settings. The biosensors‐based point‐of‐care (POC) diagnostics have shown huge potential to detect microbial pathogens in a faster, cost‐effective, and user‐friendly manner. The use of various transducers such as electrochemical and optical along with microfluidic integrated biosensors further enhances the sensitivity and selectivity of detection. Additionally, microfluidic‐based biosensors offer the advantages of multiplexed detection of analyte and the ability to deal with nanoliters volume of fluid in an integrated portable platform. In the present review, we discussed the design and fabrication of POCT devices for the detection of microbial pathogens which include bacteria, viruses, fungi, and parasites. The electrochemical techniques and current advances in this field in terms of integrated electrochemical platforms that include mainly microfluidic‐ based approaches and smartphone and Internet‐of‐things (IoT) and Internet‐of‐Medical‐Things (IoMT) integrated systems have been highlighted. Further, the availability of commercial biosensors for the detection of microbial pathogens will be briefed. In the end, the challenges while fabrication of POC biosensors and expected future advances in the field of biosensing have been discussed. The integrated biosensor‐based platforms with the IoT/IoMT usually collect the data to track the community spread of infectious diseases which would be beneficial in terms of better preparedness for current and futuristic pandemics and is expected to prevent social and economic losses.

**Abbreviations**
AI: artificial intelligence
AIV: avian influenza virus
BAW: bulk acoustic wave
BDD: boron‐doped diamond
BLM: bilayer lipid membrane
CdS QDs: cadmium sulfide quantum dots
CNT‐PPy: carbon nanotube‐polypyrrole nanocomposite
COVID‐19: coronavirus disease
CRISPR: clustered regularly interspaced short palindromic
CSV: classical swine fever
DEDTC: diethyldithiocarbamate
DENV: dengue virus
EVD: Ebola virus disease
EWI: evanescent wave interferometer
FA: fluorescent‐labeled antibody method
FET: field‐effect transistor
FO: fiber optic
FRMB: Fresnel reflection microfluidic biosensor
HBe Ag: hepatitis B e antigen
HBsAg: hepatitis B surface antigen
HCR: hybridization chain reaction
HCVcAg: hepatitis C core antigen
HIV: human immunodeficiency virus
HPV: human papillomavirus
IMAS: immuno‐magnetic assay system
InN: indium nitride
IoMT: internet of medical things
IoT: internet of things
MB: magnetic beads
MB: methylene blue
MBs: magnetic bead
MDR: multidrug‐resistant
MERS: middle east respiratory syndrome
MIPs: molecularly imprinted polymers
ML: machine learning
mMTC: massive machine type communications
MNPs: magnetic nanoparticles
NAATs: nucleic acid amplification tests
Nov: norovirus
Nov‐LPs: norovirus‐like particles
NP: nucleoprotein
PB: Prussian blue
PCR: polymerase chain reaction
PdCu TP: PdCu tripod
PDMS: polydimethylsiloxane
PEC: photoelectrochemical
PG: porous graphene
PNA: peptide nucleic acid
POC: point‐of‐care
POCT: point‐of‐care testing
PRRSV: porcine reproductive and respiratory syndrome virus
QCM: quartz crystal microbalance
QD@MHS NPs: quantum dots inside iron oxide magnetic hollow sphere nanoparticles
QDs: quantum dots
QoS: quality of service
RCA: rolling circle amplification of amplicons
RM: resonant mirror
SARS: severe acute respiratory syndrome
SARS‐CoV‐2: severe acute respiratory syndrome coronavirus
SNPs: single‐nucleotide polymorphisms
SPE: screen‐printed electrode
SPGE: screen‐printed graphene electrodes
SPR: surface plasmon resonance
SWCNT: single‐walled carbon nanotube
SWV: square wave voltammetry
TGA: thioglycolic acid
UPLC: ultra‐performance liquid chromatography
uRLLC: ultra‐reliable and low‐latency communications
VONP‐LPs: V_2_O_5_ nanoparticles‐encapsulated liposomes
ZIKV: Zika virus
μ‐PADs: microfluidic paper‐based analytical devices

## 1 INTRODUCTION

Any deviation from a condition of good health and well‐being caused by infectious agents is called infectious disease. Infectious agents include pathogenic microorganisms such as bacteria, fungi, viruses, or parasites.^1^ Infectious diseases may be spread directly or indirectly from one person to another. The spread of infectious diseases usually occurs when the infected person during their incubation period touches, coughs or sneezes near someone who is not infected or exchanges body fluids while having sexual contact.^2^ The incubation period is the period during which the infected person can infect the non‐infected, healthy individuals.^3^ Sometimes, the infected person may not show the symptoms but can infect healthy individuals.^4^ Another direct spread of infectious diseases occurs from mothers to their children either through the placenta for the unborn child or through breast milk.^5^ The indirect transmission of infectious diseases occurs if the microorganism remains on physical objects such as doorknobs. When an infected person touches the object, they left germs that in contact with the healthy person can cause infection.^6^ Infectious diseases are also spread indirectly via vectors, the most common vector is the mosquito. The mosquito can cause and spread the diseases such as dengue, and malaria.^7^, ^8^ Zoonotic diseases have also come under infectious diseases. Zoonotic diseases are animal diseases which when transmitted to humans can cause infectious diseases. Some of the zoonotic diseases include rabies, toxoplasmosis, leptospirosis, campylobacter, and swine flu. An estimation of 60% of infectious diseases is zoonotic diseases.^9^, ^10^

Pandemics caused due various infectious diseases are always a concern. In the past, bacterial pandemics have caused a lot of calamities. Cholera was one of the most concerned bacterial infections. The first cholera pandemic was caused in 1817, which was followed by the second, third, and many cholera pandemics till now.^11^ The flea‐borne bacteria Yersinia pestis, which caused the third plague, the Black Death, and at least three more human plague pandemics, is what causes plague (Zietz and Dunkelberg, 2004). Furthermore, a sizable section of the population is increasingly becoming concerned about their health due to the spread of various infectious diseases (such as malaria, cholera, and tuberculosis) to wide geographic areas.^11^ Infectious organisms, such as Yersinia pestis, Bacillus anthracis, and the variola virus, have the potential to be employed as bioweapons and pose a threat to humanity. Contrary to this, the effects of specific fungal diseases on human health are significant and each year, there are over 220,000 new cases of cryptococcal meningitis worldwide, which cause 181,000 deaths, mostly in sub‐Saharan Africa. More than 400,000 people get Pneumocystis pneumonia and pass away without receiving treatment. One of the most prevalent opportunistic fungal diseases among HIV/AIDS patients in Latin America is histoplasmosis, which causes death in about 30%. Morbidity rates associated with fungus infections are therefore a significant health concern worldwide.^12^

The new coronavirus disease (COVID‐19) caused due to the Severe Acute Respiratory Syndrome Coronavirus (SARS‐CoV‐2) is the most recent global pandemic with 281,808,270 confirmed cases and 5,411,759 deaths up to December 29 with 1,351,175 cases in the last 24 h as reported by WHO.^13^ In history, no other viral disease has caused this much threat to human beings.^14^, ^15^, ^16^, ^17^, ^18^ The other pandemics that occurred in the past years include the 1918 influenza pandemic, the human immunodeficiency virus (HIV) infection pandemic, the SARS‐CoV pandemic in 2003, and the middle east respiratory syndrome (MERS) pandemic in 2012. All these infectious diseases had affected global health and caused an economic burden. For the prevention and control of these infectious diseases, some measures need to select. The infection can be inhibited by preventing the spread from the infection source, breaking down the chain by cutting the process of transmission, distancing from the infected patients, and changing behavior like maintaining hygiene and sanitation, using condoms, using masks, healthy diets, taking vaccines, and proper medications.^19^, ^20^, ^21^

The infectious agents invade the healthy organism through the direct and indirect routes as described. Upon the invasion of the microbial agents, the organism's immune system fights the infection and protects the organism. There are two types of immunity that play role in human organisms, namely innate and adaptive immunity. Innate immunity is germline‐encoded, so keeps invariable throughout the person's lifespan.^22^ Natural killer cells are lymphoid cell that is innate and facilitates the recognition of the infection. In contrast to birth‐innate immunity, adaptive immunity is generated by somatic cell line mutations and reorganizations. This type of immunity allows the generation of memory of the encounters made after birth and protects individuals throughout their life span. T and B cells are the two types of adaptive immunity‐associated receptors. B cells secrets the immunoglobulins, which protect the organisms from foreign attacks.^23^ The innate and adaptive responses that occur due to infection are termed cytokines. Normally, a sprinkling of cytokine is enough to protect from the infection, but when it overflows due to excessive production, can spread to the whole body. The flood of cytokines that happens upon the infection is termed a cytokine storm. Excessive production of cytokines once starts is very complex to control. It can cause disturbance of physiologic functions, can cause shock, and can even reach the stage of death. For the prevention, early diagnosis can prevent the cytokine storm.^24^, ^25^, ^26^

A person with weaker immunity is more likely to be infected by the disease. The weaker immunity can be because of age, drugs, or some other diseases like diabetes, obesity, etc. The period between the infection and the onset of symptoms is known as the incubation period, various pathogens have different incubation periods. The symptoms may be very mild to very severe symptoms. In some cases, the disease may be asymptomatic also.^27^ The diseases last until the infection lingers or the person dies. The infectious diseases can be grouped into three categories, first, the diseases which can cause a high number of deaths, second the ones which can cause a high number of individuals with disabilities, and third the severe ones which can spread rapidly and cause serious global problem.^28^

Out of these controlling methods, the source of infection is the most important. The source of the infection can be controlled by early detection, early isolation of the infected from the non‐infected, and early treatment. For fulfilling the above‐stated demand, there is a need for rapid, accurate, sensitive, and economical detection methods.^21^ The conventional techniques used for the identification and detection of various strains of pathogens mainly involve culture techniques, CT scan, ELISA, serological testing, PCR, chromatographic techniques, etc. These conventional techniques are time‐consuming, required sophisticated instruments, and need trained personnel.^29^ Due to the delayed diagnosis, there is a risk of the spread of infectious diseases from the infected ones to the non‐infected ones. The limitations of conventional techniques need to be addressed properly. With the advent of advanced biosensing approaches the loop whole of conventional diagnostic modalities can be taken care off.

Biosensors are powerful analytical tools, having the potential for application to a wide range of analytes ranging from environmental contaminants to drug discovery, and medical diagnostics to security and defenses. The biosensor combines a bio‐sensitive element also called biorecognition elements with the physicochemical transducer which is connected to a detector for the detection of specific analytes. Biosensors are economical, reliable, favorable, easy to use and portable devices with high sensitivity, and selectivity and are specific to the target analyte.^30^, ^31^, ^32^ Based on the recent advances in microbial sensing, electrochemical, optical, and microfluidics‐based techniques are gaining much attention.^33^

**FIGURE 1:**
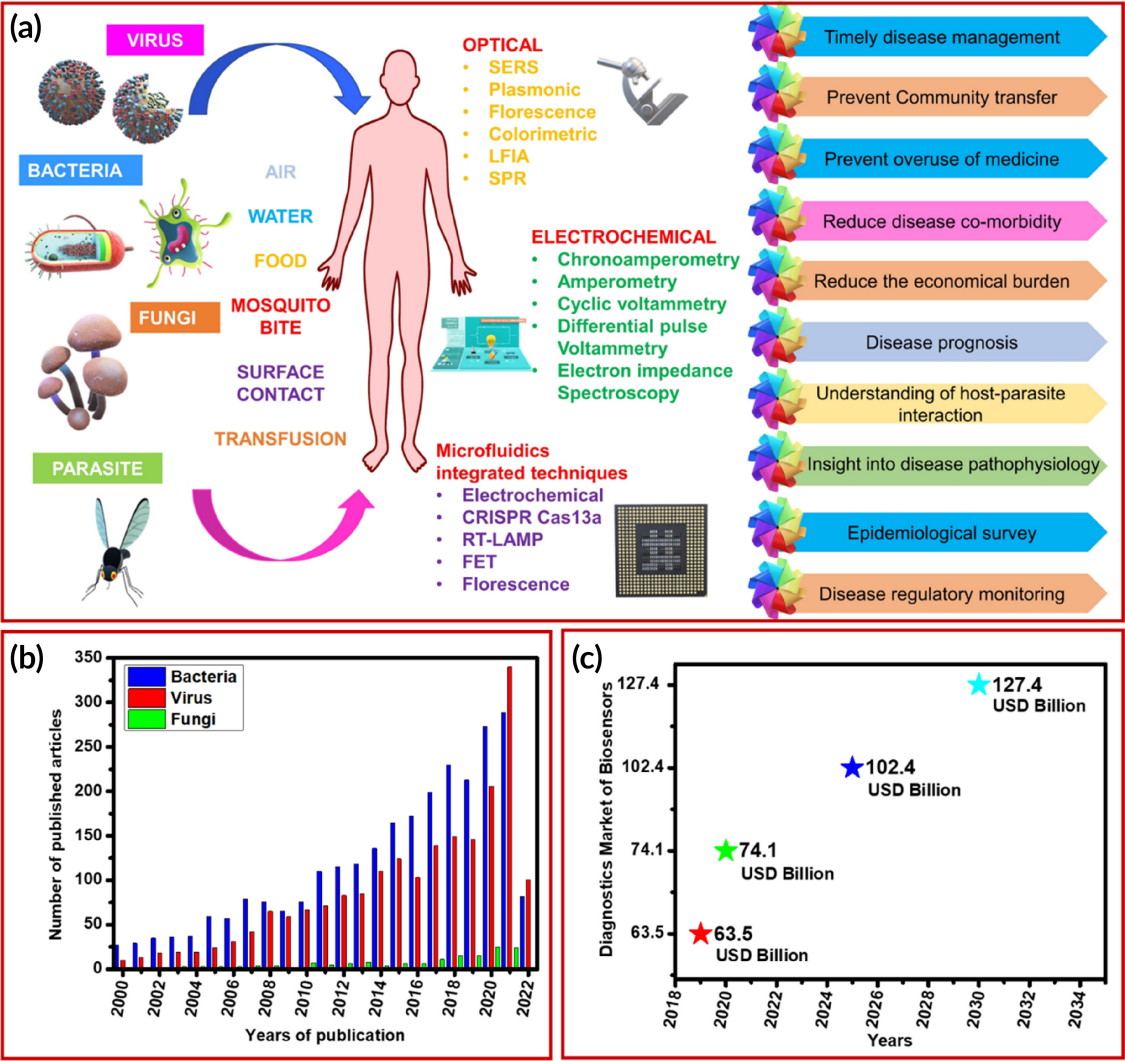
(a) Various microbial pathogens mediated disease diagnostic using optical, electrochemical, and microfluidic devices and their advantageous implication. (b) Several published papers for biosensor‐based detection of bacteria, viruses, and fungi (Data was obtained from “Web of Science” with “Biosensor for detection of bacteria, virus, fungi” entered as “Subject” in the search box [last access date: May 4, 2022]). (c) commercial market of biosensor‐based diagnostics for infectious agents.

The electrochemical techniques are an electroanalytical method of analysis that studies the electrochemical behavior of the materials used to modify the electrode conductive surface. In electrochemical‐based detection, when the specific analyte binds to the biorecognition element, it generated electrical signals, these electrical signals are then converted to quantifiable results with the help of transducers which monitor the amount of analyte present.^22^, ^34^, ^35^ Electrochemical method of analysis includes potentiometry, amperometry, and voltammetry.^36^, ^37^ The optical method of analysis includes techniques like surface plasmon resonance, interferometers, ring‐resonators, fiber‐optics photonic crystals, and planar optical waveguides. Optical biosensors track and recognize analytes by the calculation of complete reflection which senses the change in the absorbance, fluorescence, polarization, luminescence, refractive index, etc.^19^, ^38^ Microfluidics technology deals with a very little volume of samples and reagents which is why most suitable for disease diagnosis.^39^ A microfluidics‐based biosensor is an on‐chip detection that allows the detection system to be portable, disposable, and applicable for real‐time detection and can be easily integrated with any of the transducers like optical and electrochemical.^40^ Microfluidics‐based systems are speedy, economical, give high throughput, and are portable.^41^ However, the miniaturization of these biosensing platforms is a challenging task that needs special attention. Recently, portable point‐of‐care testing (POCT) devices have gained much attention in monitoring infectious diseases.^42^ The POCT devices enable rapid and early detection of diseases which can further help in early treatment and can save lives. There is considerable attention in the scientific community to the detection of microbial pathogens using optical, electrochemical, and microfluidic devices (Figure 1a) which is very well reflected by several published papers for biosensor‐based detection of bacteria, viruses, and fungi (Figure 1b). The commercial market of biosensor‐based diagnostics for infectious agents gaining sufficient momentum (Figure 1c).

The integration of these devices with the internet‐of‐things (IoT), internet‐of‐medical‐things (IoMT) and artificial intelligence (AI) can serve to monitor diseases with one click.^43^, ^44^, ^45^ The IoT is a smart solution to disease tracking and monitoring. The IoT can enable real‐time monitoring of diseases and can warn the public all around the globe.^46^ The IoT‐based devices can perform multiple tasks such as tracking the spread of disease, monitoring and responding to public healthcare, and can also use to implement effective preventive and curative measures. The AI‐integrated devices may serve many purposes like material innovation, receptors examination, signal acquisition and its transportation, processing of the data, and also the decision system.^47^

Owing to the advantages of electrochemical biosensors and their integrated platform in terms of rapid, ultra‐low detection limit and cost‐effectiveness, the design and construction of electrochemical and microfluidic biosensors for detecting microbial pathogens such as bacteria, viruses, fungus, and parasites are reviewed in this study. Biosensors integrated with the IoT and the IoMT‐‐‐ to collect data to track the spread of infectious illnesses in communities have been discussed. In the end, the difficulties encountered during the manufacture of POC biosensors were explored, as well as anticipated future improvements in the field of biosensing.

## 2 PAST, PRESENT, AND FUTURE OF BIOSENSORS FOR INFECTIOUS DISEASES DIAGNOSTICS

**FIGURE 2:**
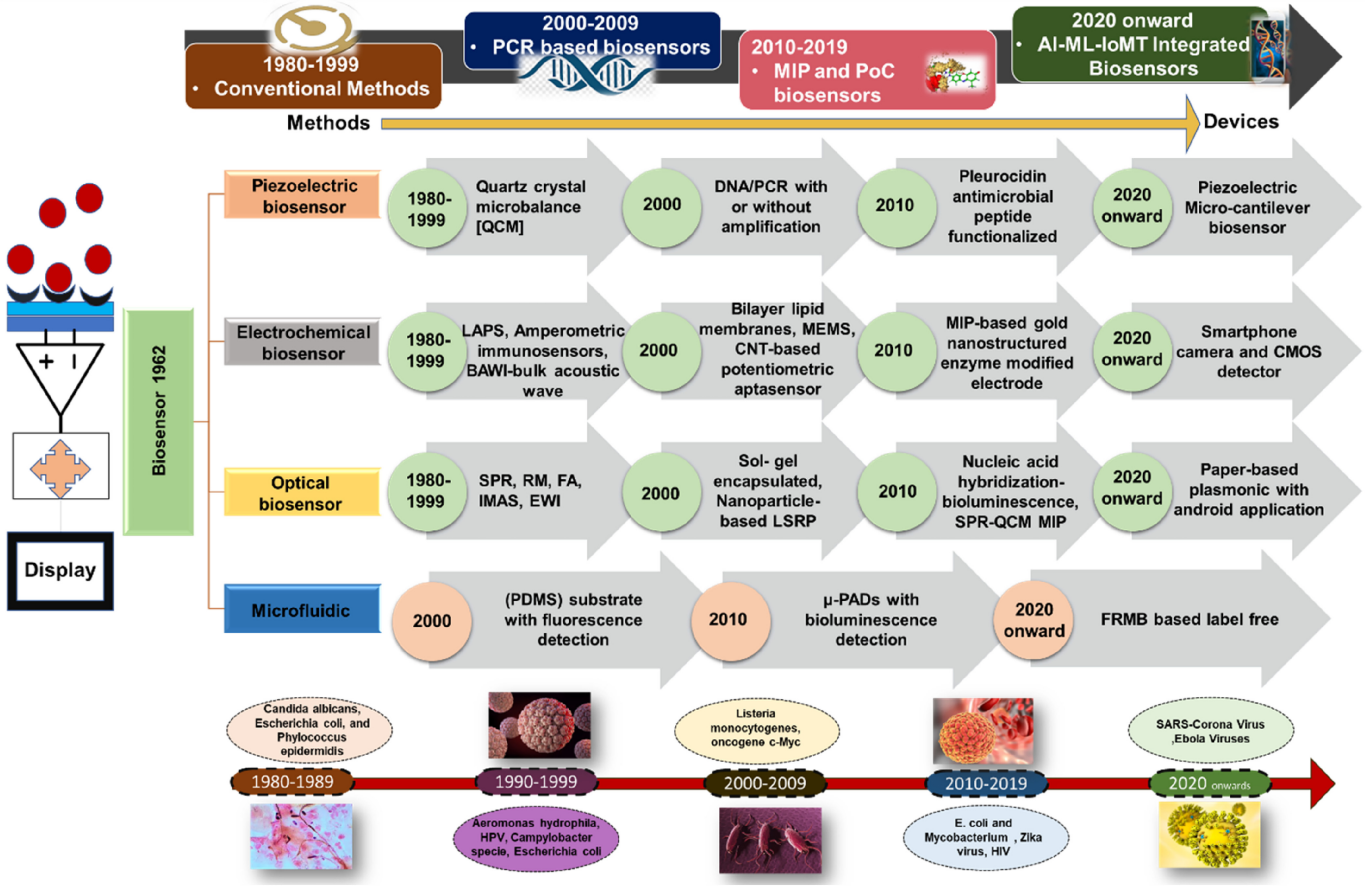
The paradigm shift in the field of development of biosensors for the detection of microbes.

Biosensors are diagnostic devices that can detect many biomolecules, they are frequently utilized for the detection of clinical pathogens like bacteria and viruses, with excellent results.^48^ Clark and Lyons initially addressed the biosensor idea in 1962 when they built an oxidase enzyme electrode for glucose detection.^49^ In biosensors for bacterial detection, biological recognition components such as receptors, nucleic acids, or antibodies are normally in intimate contact with an appropriate transducer.^50^ Biosensors are classified into four categories based on the manner of signal transmission: optical, piezoelectric, electrochemical, and microfluidic (Figure 2). With the discovery of different micro‐organisms that are present in the environment and responsible for health issues in populations around the globe, various detection techniques and bio‐sensing devices were emerging.^48^, ^50^, ^51^ Biosensors have sparked scientific study to improve the development of biosensor technologies that can transcend traditional in vitro diagnostics for illness diagnosis and health monitoring, owing to their enormous potential in medical diagnostics.^52^ A historical perspective and breakthrough discoveries in the field of development of Biosensors for the detection of microbes have been illustrated in Figure 2. In the 1980s, Conventional diagnostic techniques are well established as gold standards for the diagnosis of many infectious diseases.^53^ A technique was developed which used a thin culture medium film‐coated quartz crystal microbalance (QCM) sensor.

The Quartz Crystal Microbalance (QCM) is a highly sensitive mass balance that analyzes changes in mass per unit area at the nanogram to microgram level. Quartz is a piezoelectric substance that can be induced to oscillate at a certain frequency by providing a suitable voltage, often via metal electrodes.^54^ This type of biosensor has a low sensitivity, which prevents it from directly detecting tiny molecules. Instead, signal amplification is needed since a relatively high mass must be bound to produce a detectable change in signal.^55^, ^56^ Fiber optic (FO) biosensors are another type of biosensor that has been the subject of intense research since the early 1980s, owing to their potential sensitivity, detection speed, and flexibility to a wide range of assay conditions.^57^ The field of optical fiber biosensors is fairly broad, with several applications that have been documented in the literature, mostly through the use of evanescent wave detection.^58^ The exponential advancement in micro and nano‐level technologies leads to a new era for pathogenic detection techniques. In the 1990s, optical‐based biosensors were advancing, one of the literature reported, that a reusable Bulk Acoustic Wave (BAW)‐Impedance sensor has been created for continuous monitoring of Proteus Vulgaris development and numbers on the surface of a solid medium.^59^ One of the reported sensors is based on bacteria converting electron‐deficient or weakly charged substrates into highly charged end products, resulting in a change in medium conductance.^60^ Optical transducers are particularly appealing for use in direct (label‐free) bacterium identification. When cells connect to receptors placed on the transducer surface, these sensors may detect minute changes in refractive index or thickness.

Several optical techniques have been reported for the detection of bacterial pathogens including mono‐mode dielectric waveguides,^61^, ^62^ surface plasmon resonance (SPR),^63^ resonant mirror (RM),^64^ fluorescent‐labeled antibody method (FA),^65^ immuno‐magnetic assay system (IMAS),^66^ evanescent wave interferometer (EWI).^58^ In the late 1990s, the identification of nucleic acid led to the creation of DNA biosensors and DNA microarrays.^67^ A technique for detecting and genotyping HPV in human cervical scraping samples was also developed by one of the research groups, which combined polymerase chain reaction (PCR) with DNA piezoelectric sensors.^68^ Physical adsorption, covalent attachment, or physical entrapment are often used to immobilize recognition and signaling biomolecules (typically proteins that convert the recognition event into an optical signal) on solid substrates for solid‐state optical biosensors. In the 1900s, the synthesis of sol–gel silica material met most of the characteristics of the optical biosensors.^69^ Following this time frame, electrochemical‐based biosensors also used nano‐materials, as they provide high sensitivity to the sensor for microorganisms. Nanoparticles‐based amplification techniques have enhanced the sensitivity of bioelectronics analysis by many orders of magnitude.^70^ In 2000, one of the researchers offers a novel ion‐channel biosensor for direct and quick detection of Campylobacter species based on a supported bilayer lipid membrane. This biosensor sensing element is made up of a stainless‐steel working electrode covered by an artificial bilayer lipid membrane (BLM).^71^ Another group reported the use of microelectromechanical systems, heterobifunctional crosslinkers, and immobilized antibodies to create a biosensor for bacterial detection. The detector was composed of (100) silicon with a 2m insulating layer of thermal oxide, and it measured a change in impedance caused by bacteria trapped on interdigitated gold electrodes.

For producing the biological sensing surface, antibodies specific to *Escherichia coli (E.Coli)* were adsorbed on the oxide between the electrodes.^72^ Another group of researchers demonstrates that single‐walled carbon nanotube (SWCNT) potentiometric sensors based on aptamers are extremely selective and can be successfully utilized to identify live microorganisms in an experiment in near real‐time, making pathogen detection as simple as monitoring pH. They use this sensor to detect the different concentrations of bacteria with help of the EMF technique.^73^ Another research group uses quantum dots (QDs) as fluorescent markers to produce a quick, specific approach for detecting *Listeria monocytogenes*. QDs are semiconductor nano crystals that range in size from a few nanometers to a few hundred nanometers, and their high quantum yield makes them particularly useful in optical detection.^74^, ^75^ Advances in micro and nanofabrication methods have accelerated the transition from basic systems to lab‐on‐a‐chip prototypes. Microfluidic systems were interested in integrating sample handling, reagent mixing, and separation and detection processes.^76^, ^77^ One of the research papers developed fluorescence detection through a microfluidic biosensor module for the identification of microorganisms. A network of micro‐channels constructed in polydimethylsiloxane (PDMS) substrate made up of the microfluidic biosensor, DNA/RNA hybridization, and liposome signal amplification is used as recognition elements of biosensors.^76^

In past decades, microfluidic paper‐based analytical devices (μ‐PADs) were the new type of POC diagnostic gadget. Paper is lightweight, thin, flexible, combustible (disposable), compatible with biological samples, and chemically modifiable in the 2010s. Hydrophobic barriers establish microfluidic channels, which are patterned by infusing paper with photoresist and subjecting them to UV light.^77^ Other than microfluidic‐based biosensors, nucleic acid hybridization‐based biosensors were also being developed for pathogens such as *E. coli* and *Mycobacterium tuberculosis*. A wide range of microorganisms has been detected using bioluminescence systems.^78^ An electrochemical sensor based on graphene oxide polymers imprinted for Zika virus (ZIKV) detection was developed by one of the research groups, the biosensor was utilized to detect by monitoring changes in the electrical signal with varying viral amounts in buffer and serum. Our approach's detection limit is comparable to that of the real‐time quantified reverse transcription PCR method.^79^ Nowadays, infections are among the most catastrophic natural catastrophes, having a significant influence on global well‐being (in terms of severe morbidity and death) as well as economics. Presently, the pursuit of smart systems such as smart implants, prosthetics, and biosensors is obtaining focus because of their role in disease management and control, rehabilitation, and other post‐surgical operations.^80^ The use of nano‐chips, nano‐sensors, and nano‐robots in smart sensing and monitoring systems has the potential to monitor vital signs, drug medication, and the identification of infections linked to illnesses.^81^

Chemo‐metrics is essential in biosensor detection, analysis, and diagnosis. In recent years machine learning (ML) has made substantial advancements in the discipline of AI. However, innovative advanced ML approaches, particularly deep learning, which is well‐known for image analysis, facial recognition, and speech recognition, have remained elusive to the biosensor community.^82^ While taking potential and advances into account with AI to manage an earmarked infectious disease. Smart biosensors have the potential to aid physicians in monitoring and predicting illnesses for early intervention by sensing important parameters.^47^, ^68^, ^83^, ^84^ The artificial intelligence‐powered biosensor takes physiological and other data from patients' wearable biosensors and uses AI or ML algorithms to identify changes in their important signal patterns.^83^ IoMT‐assisted sophisticated biosensors technologies are being developed by researchers for POCT of infectious diseases like malaria, dengue fever, H1N1, HIV, human papillomavirus (HPV), and Ebola virus disease (EVD), ZIKV, and COVID‐19 infection.^47^

The combination of smartphones and nanotechnology has created smart nanosensors that might help the general population utilize a smartphone as colorimetric, fluorimetric, and electrochemical sensors.^84^ Using augmented reality which is critical for the diagnosis of COVID‐19, a paper‐based plasmonic biosensor linked with a smartphone was constructed for the automated detection of interleukin 16.^85^ The smartphones are used to identify colorimetric and fluorimetric changes, which are imaged and analyzed using a smartphone camera and a standalone android application, They can also detect electrochemical alterations, which are analyzed by using a smartphone camera and CMOS detectors for further data interpretation and communication.^85^, ^86^ One of the research groups reported a novel all‐fiber Fresnel reflection microfluidic biosensor (FRMB) was developed by merging an all‐fiber optical system, a microfluidic chip, and a multimode fiber bio‐probe. Employing the Fresnel reflection approach, both the SARS‐CoV‐2 IgM and IgG antibodies against the SARS‐CoV‐2 spike protein could be sensitively assessed in 7 min using the secondary antibodies‐modified multimode fiber bio‐probe. The detection limits for SARS‐CoV‐2 IgM and IgG were 0.82 ng ml^−1^ and 0.45 ng ^−1^ respectively.^87^

This is a simple method with significant potential for POC diagnostic platforms in the fight against COVID‐19 and other future pandemics and endemics.^88^ Differential diagnosis of pathogenic infections, such as COVID‐19 and influenza, is critical, with special emphasis paid to super‐spreading events, a likely extended incubation time, specific consequences, and therapies. A label‐free, self‐powered, and ultrafast immuno‐sensor device based on the triboelectric effect is suggested in one of the recently reported work. To detect SARS‐CoV‐2 and H1N1, equilibrium constants of particular antibody–antigen reactions are complemented with IEP‐relevant electric charges of antigens. The highest capture efficiency of 85.63 percent is attained by optimizing simulation features such as fluid flow and geometrical parameters.^89^

## 3 RECENT ADVANCES IN MICROBIAL SENSING

In the last decade, the science of biosensing has seen tremendous progress in illness diagnostics.^90^, ^91^ Drug‐resistant bacteria are surpassing drug discovery efforts, putting current antibiotics in jeopardy and threatening numerous unavoidable medical procedures which are taken for granted.^92^, ^93^, ^94^ Controlling this worldwide threat will require the invention and application of increasingly wide‐ranging diagnostics for infectious diseases. Infectious disease laboratory diagnosis is currently predominantly based on cell culture approaches that provide results in days, which hamper the decision of timely selection of effective therapeutic antimicrobials.^35^, ^90^, ^92^, ^95^ The nucleic acid amplification tests (NAATs) provide speedy results since they detect microbial NA directly in patients' samples, often taking less than an hour or two.

Contrary to this, existing NAAT technologies have two major drawbacks that have hampered their acceptance, particularly in terms of detecting the whole range of mutant variants of microbes that are drug resistant termed multidrug‐resistant (MDR) microbes.^90^, ^92^ The foremost is their limited level of multiplexing, which is referred to as the total number of sequences (or strains) that can be identified in a single reaction.^96^ The second issue is their inability to detect single‐nucleotide polymorphisms (SNPs) or other alterations/mutations due to their low resolution and precision. However, it is possible to examine localized sequence alterations (usually 3–6 nucleotides) using allele‐specific primers or fluorophore‐labeled probes, however, the exact base change and the precise coordinate cannot be obtained with high confidence.^97^ The limitations of these techniques can be well tackled using advanced biosensors which include electrochemical, and microfluidic‐based platforms, which provide cost‐effective, ultra‐highly sensitive, and selective detection of target analyte within a limited time frame. The details of electrochemical, and microfluidic‐based POCT for infectious disease diagnostics will be discussed in the next section.

### 3.1 Electrochemical‐based microbial biosensors

The detection of biomolecules via biosensors requires a chemical recognition element that captures the target analyte and the chemical energy from the biological interactions/chemical reactions would then be translated into electrical energy via a physio‐chemical transducer. Further, this signal needs to be sent to a detector for signal processing and analysis. In the case of an electrochemical biosensor, an electrochemical transducer is used on a chemically modified electrode that combines with a biological analyte for selective detection of a target analyte.^98^, ^99^ Among the numerous merits of electrochemical biosensors, the ability of high sensitivity, low detection limit, portability, high stability, ease of operation, and high‐throughput diagnostics which are most demanding in recent times for POCT. Microbial diseases encompass either viruses, bacteria, parasites, or fungi which are of huge interest owing to their high mortality rate and severity.

**FIGURE 3:**
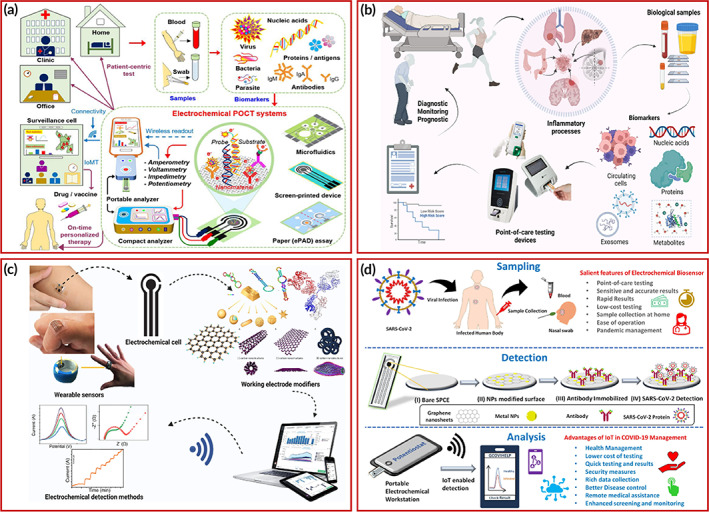
(a) Pictorial illustration of IoMT integrated electrochemical POC testing devices. Copyright from Reference 100. (b) IoMT‐based POC monitoring, treatment, and management for Inflammation based diseases, (c) graphical representation of working methodology of electrochemical platforms in POC settings. Copyright from Reference 101. (d) Graphical interpretation of sampling, detection, and analysis of graphene‐based IoT integrated electrochemical biosensor. Copyright from Reference 107.

POCT is a fundamental requirement for an early diagnosis for diseases concerning contact and non‐contact‐based transmissions. The screening and diagnosis of the patients need to make localized and personalized enabling better and faster management prospects. Figure 3a represents the linking between electrochemical‐based POC platforms and monitoring methods that helps report disease statistics throughout an outbreak situation. The advantages of IoMT‐based POCT include cheap, simple, user‐friendly devices without skilled persons that can be performed at clinics, houses, workplaces, etc. Nonetheless, IoMT‐based POC tests support to fulfill high‐demand screening and high‐throughput testing at a global scale reducing the burden at centralized facilities and enabling easy monitoring and fast diagnostics.^100^

Several pathogenic diseases are primarily responsible for inflammation, giving rise to various inflammation biomarkers. Such biomarkers are characterized and effectively diagnosed by IoMT‐integrated POC devices for efficient, swift, accurate large‐scale disease monitoring. Electrochemical‐based POC devices for the detection of such microbial disease is systematically discussed in this section. Figure 3b illustrates the diagnosis and POC‐based monitoring of inflammatory diseases for modernized healthcare governance.^101^

Electrochemical biosensors are quite versatile and can be used for the accurate, early, swift, and cost‐effective diagnostics of bacterial diseases,^102^ tropical diseases,^103^ infectious viral diseases,^104^ and pathogen detection,^105^ respiratory virus detection,^106^ etc. In the view of the characteristics of electrochemical biosensors, the development of IoMT‐based POC devices would drastically boost the current requirement of global, large‐scale, cheap, quick, and sensitive detection of the disease biomarkers. Figure 3c demonstrates the peculiar attributes of electrochemical biosensors in terms of its substrate modification, use of nanomaterials, high sensitivity, advancements to improve shelf‐life, reduce biofouling, multi‐plex detection ability, digitalization, and ease of integration with other techniques. The use of IoMT‐based platforms goes a long way to solve current issues with increasing demands. Electrochemical biosensors preclude advantages of miniaturization, portability, selectivity, and POCT possibilities that promote its usage in clinical settings even at a global scale.^101^ Detection of analyte by employing graphene‐based IoT integrated electrochemical biosensor has been shown in Figure 3d.^107^

#### 3.1.1 Detection of viruses using electrochemical biosensors

The past and the present outbreak of viruses alarmed researchers for the development of electrochemical biosensors for the detection of infectious agents. The rapid and sensitive detection of viruses is the need of the hour since the global population has been affected by the COVID‐19 pandemic.^108^ Various viruses have been responsible for adversely affecting human health by degrading the quality of life. The probable solution to such infectious diseases is the development of effective diagnostic tools such as electrochemical biosensors. For instance, recently, Silva et al.^109^ detected non‐structural protein (NS2B) of ZIKV through a screen‐printed electrode (SPE) based immunosensor. They employed the amperometry detection technique by utilizing the redox catalytic activity of Prussian blue (PB) embedded into carbon nanotube‐polypyrrole nanocomposite (CNT‐PPy). They showed the specificity of the electrochemical sensor with dengue (DENV) samples. The no‐probe PB@CNT‐PPy‐based immunosensor was rapid and convenient that increased the sensitivity to 98%.

In another study, Cifti et al.^110^ detected the Ebola virus by fabricating an electrochemical genosensor consisting of rolling circle amplification of amplicons (RCA) in nucleic acid as RCA products (RCPs). Further, magnetic beads (MB) were decorated on RCPs and later bounded to GOx by streptavidin‐biotin. Glucose after enzymatic catalysis, the transducer matrix can detect the Ebola DNA target indirectly. Synthetic Ebola samples were detected in the range of 1–100 pM as well as the proof‐of‐concept was confirmed on real Ebola patients' samples having a viral load of C_t_ = 16.

Tan et al.^111^ detected hepatitis B e antigen (HBe Ag) by an electrochemical immunosensor. They used PdCu tripod (PdCu TP) modified porous graphene (PG) (PdCu TPs/PG) nanoenzymes for the fabrication of a label‐free immunosensor. The fabricated immunosensor showed good stability within 5 weeks, having linearity ranges from 60 to 100 fg ml^−1^ and LOD of 20 fg ml^−1^. They evaluated the recovery rate in synthetic human serum samples for HBe Ag which was reported to be from 99.8% to 100.48%.

Recently, Boonkaew et al.^112^ reported a chronoamperometry technique for the simultaneous detection of hepatitis B surface antigen (HBsAg) and hepatitis C core antigen (HCVcAg) by fabricating an automated fast‐slow paper‐based platform. They fabricated an ePAD device assembly by a wax printing method. They used an in‐house fabrication technique for screen‐printed graphene electrodes (SPGE) where carbon ink was used for the working electrode. They detected the viruses in the range of 0.001–250 ng ml^−1^ for HCVcAg and 0.1–250 ng ml^−1^ for HBsAg having a limit of detection of 18.2 pg ml^−1^ for HBsAg and 1.19 pg ml^−1^ for HCVcAg. Further, they did real sample analysis in clinical serum samples. The obtained results propose that the developed platform can be an effective alternative to POCT in clinical trials and large‐scale diagnoses.

In another study by Ganganboina and group,^113^ they reported a unique dual modality electrochemical/fluorescence‐based sensor that consists of CdSeTeS quantum dots inside iron oxide magnetic hollow sphere nanoparticles (QD@MHS NPs). The synthesized QD@MHS NPs showed fluorescence, and conductivity with effective sensing when used on Au/rGO, modified electrodes. They detected HEVs from cell culture supernatant and fecal specimens of HEV‐infected monkeys. The linear range for HEV‐LPs was from 10 fg ml^−1^ to 10 ng ml^−1^ and LOD was reported to be 1.2 fg ml^−1^. Whereas the detection results of Nov‐LPs and Nov in clinical samples remarked the LOD of Nov to be 69 RNA copies ml^−1^. Furthermore, Guo et al.^114^ detected norovirus (Nov) by a photoelectrochemical biosensor (PEC). Thioglycolic acid (TGA) was used to stabilize cadmium sulfide quantum dots (CdS QDs) as a substrate on ITO electrodes to fabricate an immunosensor. They measured photocurrent to detect recombinant VP1 NoV protein by the developed PEC biosensor. Within 30 min of testing time, the LOD obtained was 4.9 pM for synthetic samples and 46 copies μl^−1^ in real samples.

**FIGURE 4:**
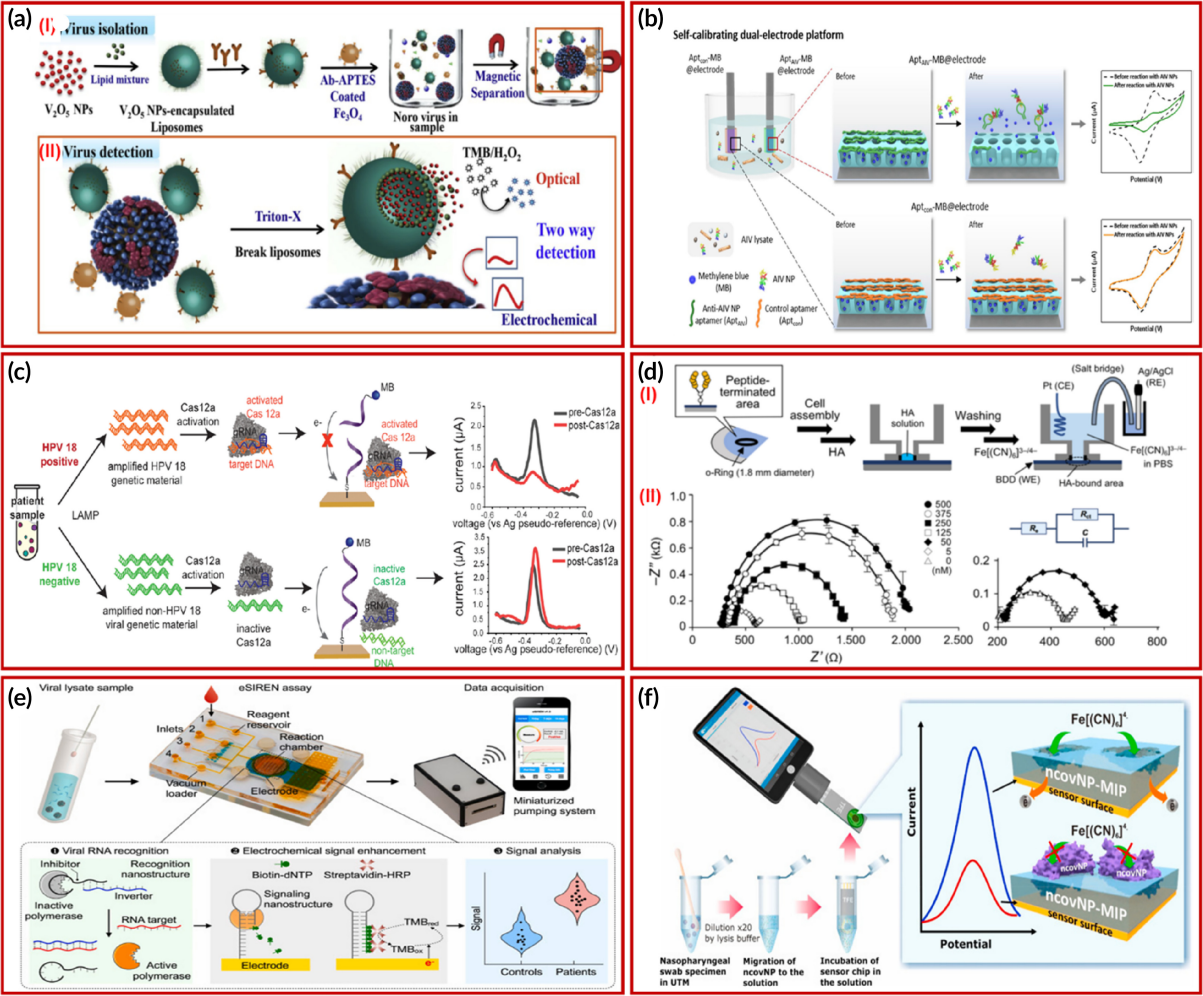
(a) Various electrochemical biosensors for the detection of different virus infections: (i) Fabrication process illustration of V_2_O_5_ NPs‐encapsulated liposomes (VONP‐LPs) and (II) detection of Nov. Copyright from Reference 115. (b) Porous nano silica‐based electrochemical aptasensing self‐calibrating platform. Copyright from Reference 117. (c) Selective binding of HPV 18 DNA with Cas12a. Copyright from Reference 118. (d) BDD electrodes for HA detection. (I) Graphical representation of BDD electrodes assisted HA measurement. (II) Corresponding H1 HA detection is represented by Nyquist curves. Copyright from Reference 119. (e) depiction of microfluidics integrated advanced electrochemical sensor for SARS‐CoV‐2 detection using molecular nanostructures Copyright from Reference 125. (f) working of an IoMT coupled electrochemical POC device to detect SARS‐CoV‐2 infection from nasopharyngeal swab specimens of patients. Copyright from Reference 126.

Ganganboina and group,^115^ developed a sensing platform having dual‐modality using V_2_O_5_ nanoparticles‐encapsulated liposomes (VONP‐LPs) for the detection of norovirus‐like particles (Nov‐LPs). The platform has both colorimetric as well as electrochemical sensing for both qualitative and quantitative results. The linear range of detection was reported from 10 fg ml^−1^ to 10 pg ml^−1^ along with the LOD of 4.1 fg ml^−1^. For clinical samples, the LOD was found to be 72 RNA copies ml^−1^. The results suggested that the dual‐modality biosensor can be effectively used in the public monitoring of various infectious diseases. The NoV detection principle and process methodology are systematically presented in Figure 4a.

Recently, Idili et al.,^116^ detected SARS‐CoV‐2 through an electrochemical aptamer‐based (EAB) sensor. For this, they used 1C and 4C as two DNA aptamers that identified the RBD of SARS‐CoV‐2 spike protein. The results validated the sensor in clinical serum as well as saliva samples within minutes. The clinical range of detection was achieved by the reported sensor.

In a recent study, Lee et al.^117^ detected the avian influenza virus (AIV) by an electrochemical aptasensor. They fabricated a dual‐electrode system using porous silica film on tungsten rods (3DNRE) by self‐assembly. They reported the detection of AIV nucleoprotein (NP) ranges from 2 nM to 2 μM having a limit of detection of 1.13 nM. The fabricated platform showed superior stability in signals (relative standard deviation, RSD: 5.86%) in comparison to the standard single‐electrode platform (RSD: 30.13%). The working principle of the self‐calibrating autosensing platform is dependent on both AptAIV and Aptcon interaction with the target analyte. The response in aptamer‐based detection depends on the capped AptAIV on silica nanostructure causing the release of methylene blue (MB), whereas Aptcon acts as a baseline that does not react with the target molecule. Corresponding redox current measurements are shown in Figure 4b.

In another study, Zamani et al.,^118^ reported an integrated platform to detect human papillomavirus (HPV) via gold leaf electrodes. They blended electrochemical with loop‐mediated isothermal amplification and a CRISPER‐based assay, which enables a lower LOD of 10^4^ total copies from cervical clinical samples. This combination has provided 100% sensitivity and 89% specificity in clinical samples. The binding of Cas12a with HPV 18 DNA cleaves MB, resulting in a decrease in its signal response, and alternatively MB signal remains the same when Cas12a does not bind with non‐specific target molecules. This mechanism is shown in Figure 4c.

Moreover, Matsubara et al.^119^ developed a boron‐doped diamond (BDD) biosensor for the detection of the human influenza virus. They used a sialyl oligosaccharide mimicking peptide to bind specifically with hemagglutinins. These modified BDD electrodes showed exceptional LOD of <1 pfu for H1N1 and H3N2 viruses. The schematic detection of hemagglutinin (HA) using the BDD electrode is given in Figure 4d.

Recently, Li et al.^120^ reported a microfluidic paper‐based analytical device (μPADs) where zinc oxide nanowires (ZnO NWs) were used to tailor the working electrode. The μPADs were used to detect a p24 antigen as a marker for HIV by employing the EIS technique. They did an optimization study for different EIS parameters as well as various ZnO NW lengths, results suggested that thick‐long nanowires have a better LOD of 0.4 pg ml^−1^ than others. They even demonstrated the ability of the sensor to detect SARS‐CoV‐2 antibodies which expand the future possibilities of the sensor.

Li et al.^121^ reported a multichannel detection of SARS‐CoV‐2 and influenza A(H1N1) virus on an electrochemical immunosensor (MEIA) via disposable SPCE. The MEIA consisted of eight channels on a single array that integrated capture and detection antibodies for SARS‐CoV‐2 and A(H1N1) simultaneously. The linear range and LOD of the immunosensor for A(H1N1) and SARS‐CoV‐2 spike protein are 4–64 unit ml^−1^, 0.15–100 ng ml^−1^, and 1.12 unit ml^−1^, 0.15 ng ml^−1^, respectively. This multichannel can potentially be used in POCT of various infectious diseases soon. The catalytic response after adding TMB and H_2_O_2_ is recorded by an HRP‐labeled detection antibody complex, which recognizes the target SARS‐CoV‐2 or A(H1N1) virus in the sample.

A dual detection platform has been reported for the detection of SARS‐CoV‐2 antigen as well as antibodies on GCEs which has the capability to be translated to SPEs for portable and POC devices. The authors reported the dual detection platform based on GO‐Au hybrid nanocomposite having a detection limit of 3.99 ag ml^−1^ and 1.0 fg ml^−1^ for SARS‐CoV‐2 nucleocapsid antigen and antibodies, respectively.^122^ Additionally, Yadav et al. reported the transition‐metal‐based nanocomposite for the detection of SARS‐CoV‐2 nucleocapsid protein. The molybdenum disulfide nanosheets decorated with polydopamine (MoS_2_‐PDA) based immunosensor showed a LOD of 2.80 ag ml^−1^ in buffer samples. The platform was even validated to detect SARS‐CoV‐2 infection in nasopharyngeal swab specimens of COVID‐19 patients.^123^ Furthermore, chitosan‐functionalized titanium dioxide nanoparticles (TiO_2_‐CS bio‐nanocomposites) modified glassy carbon electrodes have been studied for the fabrication of electrochemical immunosensor for the detection of SARS‐CoV‐2 antibodies with a remarkable enhancement in detection limit up to 3.42 ag ml^−1^ in buffer samples.^124^

Zhao et al. presented a consolidated electrochemical system integrating reconfigurable enzyme‐DNA nanostructures (eSIREN) for the detection of RNA specific to SARS‐CoV‐2. The system activates target molecules via microfluidics and nanostructures to get an enhanced electrochemical response. The eSIREN selectively combines specific RNA and does a catalytic enhancement only after hybridization and discrete target‐influenced reactions. The microfluidics helps to coordinate the connection between molecular dynamics and electronics. The integration of techniques supports automation, ease functioning, and provides automation. The system can detect target RNA lowest up to seven copies per μl, and the detection time is <20 min in real patient samples. The system has also been validated against lysed swab samples of COVID‐19 patients. The methodology and integrated assembly of the electrochemical sensor is shown in Figure 4e.^125^

Raziq et al. reported a new route by using molecularly imprinted polymers (MIPs) for the first time to detect SARS‐CoV‐2 nucleoprotein. The use of a disposable chip modified with MIP gives the sensor improved selectivity, stability, easy use, and portability. The linearity of the sensor was reported to be 111 fM capable of detecting the lowest concentration up to 15 fM. The authors also reported real swab sample results to validate the working of the system for COVId‐19 positive patients. The illustration of a rapid and portable electrochemical device is presented in Figure 4f.^126^

#### 3.1.2 Detection of bacteria through electrochemical biosensors

Bacterial infection has long been dreaded the mankind and researchers developed several electrochemical biosensors for the detection of various types of infection. One of the most vastly monitored microbial agents is bacteria, which need detection at an early stage with high sensitivity and accuracy. The proper diagnosis of bacterial microbes and implantation of effective treatment approaches for various bacterial infections is the key requirement. The use of electrochemical biosensors has been extensively exploited for the detection of numerous bacteria. For example, Kim et al.,^127^ developed an erythrocyte‐camouflaged biosensor (ECB) that detected α‐hemolysin (Hla), which is secreted by *Staphylococcus aureus* (*S. aureus*). The clinical samples were collected from human serum and EIS was used to detect Hla ranges from 0.0001 to 1 mg ml^−1^ with the LOD of 1.9 ng ml^−1^. The stability of the sensor was studied for 35 days with 99% accuracy.

**FIGURE 5:**
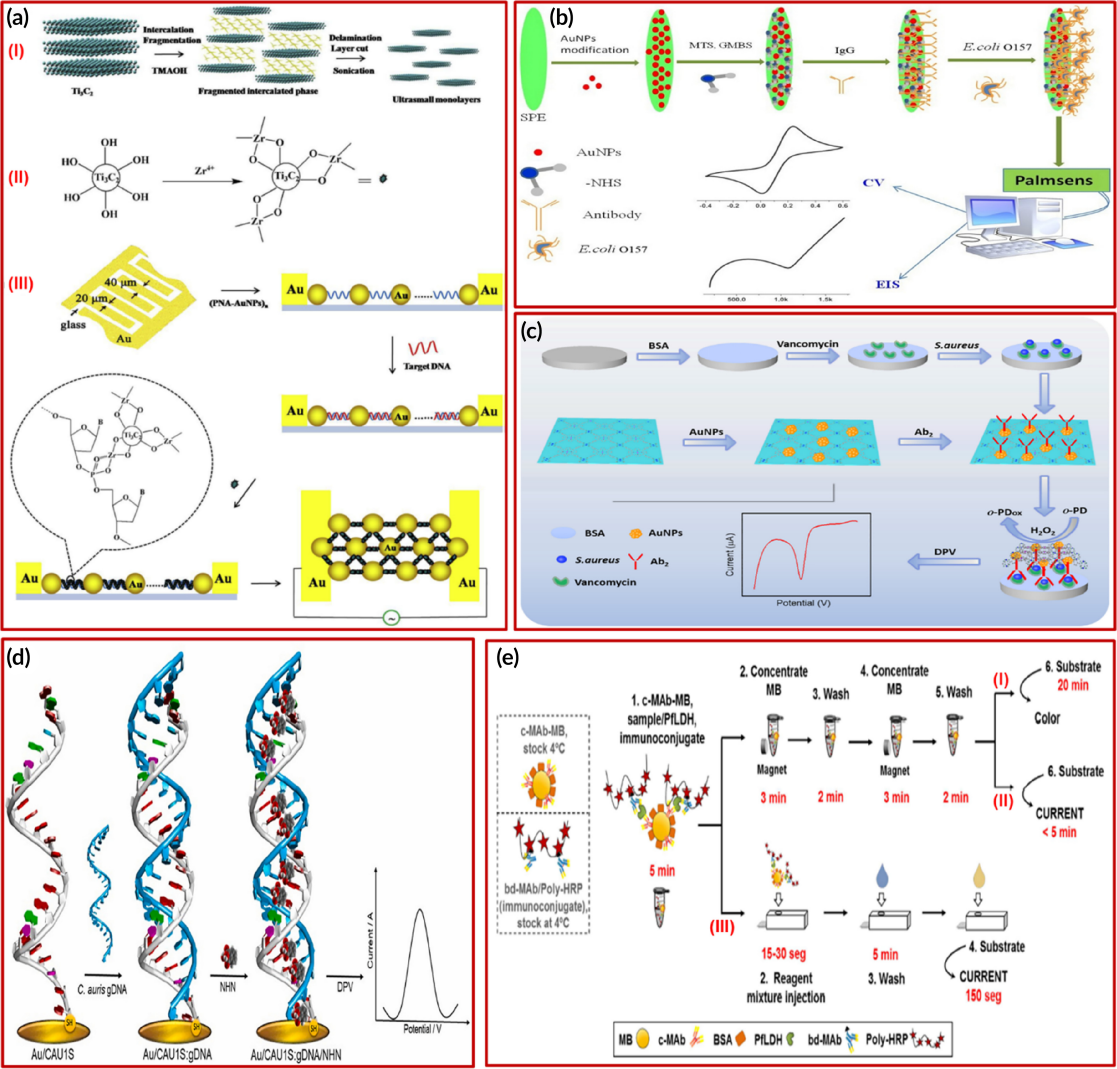
Various electrochemical biosensors for the investigation of different bacterial infections. (a) (I) Synthesis of Ti_3_C_2_ nanosheets. (II) The reaction between Ti_3_C_2_ nanosheets and ZrOCl_2_. (III) The constructed strategy of the sensor for rapid detection of M. tuberculosis Copyright from Reference 128. (b) Determination of *E. coli* O157 bacteria by AuNPs‐modified carbon SPEs. Copyright from Reference 130. (c) Graphic of MOF Nanozyme‐based detection of *S. aureus*. Copyright from Reference 132. (d) Ninhydrin assisted detection of *C. Auris* gDNA by electrochemical genosensor. Copyright from Reference 134. (e) Various arrangements of magneto‐immunoassay. (I) color‐based detection. (II) amperometric detection. (III) Different steps of detection on a chip at MP‐dsSPCE. Copyright from Reference 138.

Zhang et al.^128^ fabricated a zirconium‐cross‐linked Ti_3_C_2_ MXenes‐based electrochemical biosensor for the detection of *Mycobacterium tuberculosis* (*M. tuberculosis*). The Au NPs on the electrodes were covered by the network of hybridized target and Ti_3_C_2_ MXenes making it conductive for detection. The peptide nucleic acid (PNA) along with a specific fragment of 16 S rDNA were hybridized on the electrode substrate enabling the linking of phosphate groups of target molecules and zirconium‐cross‐linked Ti_3_C_2_ MXenes. The electrochemical analysis showed that the detection of *M. tuberculosis* was done in the range of 1 × 10^2^ to 1 × 10^8^ CFU ml^−1^. The detection was done under 2 h at a limit of 20 CFU ml^−1^. The fabrication of the biosensor for the detection of *M. tuberculosis* is shown in Figure 5a.

Similarly, Tai et al.^129^ fabricated a laser‐scribed graphene nanofiber (LSG‐NF) modified with silver nanoparticles (Ag NPs) and oil palm lignin biosensor for the detection of *M. tuberculosis* DNA. The hybridization and immobilization of target DNA on the electrode surface were confirmed through Raman, X‐ray photoelectron spectroscopy (XPS), and Fourier‐transform infrared (FTIR). They utilized electrochemical impedance for detecting target DNA up to 1fM. The stability studied showed only 10% degradation throughout 8 weeks of measurement.

In a study by Vu et al.,^130^ they used a gold nanoparticle (Au NPs) modified SPE immunosensor to detect *E. coli*. They reported a detection time of 30 min in the detection range of 10–10^6^ CFU ml^−1^ for the label‐free immunosensor having the limit‐of‐detection (LOD) of 15 CFU ml^−1^. The corresponding SPE‐based electrochemical biosensor for *E. coli* is depicted in Figure 5b.

For the detection of *Corynebacterium diphtheria* bacteria, Ziółkowski et al.,^131^ reported a diethyldithiocarbamate (DEDTC) based electrochemical immunosensor. They proposed two mechanisms of detection that can explain the electrochemical results. The first method square wave voltammetry (SWV) showed the LOD of 5 × 10^−6^ μg ml^−1^ and the second method were amperometry, which resulted in a LOD of 5 × 10^−4^ μg ml^−1^. The real human saliva diluted, and spiked samples confirmed the high selectivity of the immunosensor for diphtheria toxoid to be 0.01 Lf ml^−1^.

Hu et al.^132^ demonstrated a nanoenzyme‐amplified metal–organic framework (MOF) based electrochemical biosensor for the detection of *S. aureus* bacteria. The 2D MOF was synthesized from copper nitrate, trifluoroacetic acid, and polyvinylpyrrolidone. The Au electrodes were modified by 2D MOF, vancomycin, and anti‐*S.aureus* antibody to fabricate the immunosensor, for specific detection of *S.aureus*. The catalytic activity of nanoenzyme‐based immunosensor was studied, along with its electrochemical parameter optimization for time, pH, temperature as well as different vancomycin concentrations. The results revealed the LOD of the sensor to be six colony‐forming units CFU ml^−1^ in the detection range of 10–7.5 × 10^7^ CFU ml^−1^. The MOF nanoenzyme‐assisted detection of *S. aureus* is presented in Figure 5c.

Recently, Balayan et al.^133^ reported the detection of serum amyloid A (SAA) biomarkers of Neonatal septicemia bacterial infection. The multi‐walled carbon nanotubes (MWCNTs), manganese oxide nanospheres (MnO_2_ NSs), and cobalt oxide nanoparticles (Co_3_O_4_ NPs) were further modified by molecularly imprinted polymers (MIPs) were utilized for the fabrication of the electrochemical electrode. The detection of SAA was done using the EIS technique in the linear range of 0.01 pM to 1 μM and LOD of 0.01 pM.

#### 3.1.3 Detection of fungi by electrochemical biosensors

The robust fungal infection if not diagnosed early can cause severe health related co‐morbidities. Owing to advantages of electrochemical biosensors, several biosensors have been developed for detection of fungi. The fungal infection can be avoided by rapid, sensitive, and accurate detection through a reliable biosensor. Electrochemical biosensors are being developed with special emphasis on various microbial agents, some of which are discussed below. Particularly, to determine a fungus, Guedes et al.^134^ recently constructed a genosensor‐based novel DNA hybridization indicator. The Au electrode was modified with a specific 25‐base DNA capture probe that was able to detect *Candida auris* (*C. auris*) gDNA. They utilized DPV and EIS to detect *C. auris* in a urine sample in the range of 45 ng μl^−1^ to 4.5 pg μl^−1^. The genosensor retained high shelf life of up to 80 days with good selectivity and regeneration of signal with no loss up to 8 cycles of use. The schematic of the first genosensor for *C. Auris* is shown in Figure 5d.

In another study by Yun and group,^135^ they developed an electrochemical hydrogen peroxide (H_2_O_2_) sensor and measured the residual H_2_O_2_ after enzymatic degradation. The sensor was fabricated using palladium nanoparticles/carbonized bacterial cellulose nanocomposites, having good stability of fungal growth detection for 5 days. The study found that there is a positive correlation between catalase (CAT) and Aflatoxin B_1_ (AFB_1_). The linear range of detection for mycelia was over 0.5–3.5 U ml^−1^ and a LOD of 0.434 U ml^−1^ was reported.

For the detection of *Ochratoxin* A (OTA), Wang et al.^136^ constructed an electrochemical aptasensor consisting of gold nanoparticles modified molybdenum oxide (AuNPs‐MoOx) nanocomposite, hybridization chain reaction (HCR), and a constraint endonuclease (Nb.BbvCI)‐assisted walker DNA. The signal amplification was done by using HCR at methylene blue (MB) on silver nanoparticles (AgNPs). The electrochemical analysis was done by DPV, and the results were optimal in the range of 0.01–10,000 pg ml^−1^ with a LOD of 3.3 fg ml^−1^. Further cross‐validation in red wine apple, orange juice, and blood serum was done by ultra‐performance liquid chromatography (UPLC).

Recently, a dual‐target detection of mycotoxins was reported by Han and the team.^137^ They modified the electrode surface by simultaneous reduction of rMoS_2_‐Au and aptamers. To have a simultaneous, selective, and sensitive detection of zearalenone (ZEN) and fumonisin B1 (FB1), thionine (Thi), and 6‐(Ferrocenyl) hexanethiol (FC6S) were used as detection probes. The aptasensor showed linear detection in the range of 1 × 10^−3^ to 10 ng ml^−1^ and 1 × 10^−3^ to 1 × 10^2^ ng ml^−1^, and LOD of 5 × 10^−4^ ng ml^−1^ for ZEN and FB1, respectively. The results were validated in real maize samples as well.

#### 3.1.4 Detection of the parasite by electrochemical biosensors

Fabrication of electrochemical biosensors for detection of parasitic infection gained considerable attention due to added advantages. The diverse applicability of electrochemical biosensors has been explored for the detection of a parasite such as in a study by Ruiz‐Vega et al.,^138^ *Plasmodium falciparum* LDH (PfLDH) is detected using a POC based electrochemical magneto‐immunosensor. They used magnetic beads modified by capture antibodies (c‐MAb‐MB) and detection antibodies (bd‐MAb/Poly‐HRP). They constructed a microfluidic paper double‐sided SPCE (MP‐dsSPCE) for single use. They used an amperometric electrochemical technique to detect PfLDH in lysed whole blood. The results evidenced rapid results within 20 min, with LOD of 2.47 ng ml^−1^ in spiked samples and 200 ng ml^−1^ in lysed blood samples. Comparative to other rapid diagnostic tests, they hypothesized their device to have the least user interference and more ease of testing. The magneto‐immunoassay with various formats is mentioned in Figure 5e. Table 1 represents the tabular outline of various microbial infections detected through electrochemical biosensors.

**TABLE 1:** Electrochemical biosensors for determination of various infectious diseases

S. no.	Type of microbial organism	Sensing technique	Target analyte	Material matrix	Lod/sensitivity	References
	Virus					
1.	Zika Virus (ZIKV)	Amperometric	NS2B Protein	Prussian blue‐Carbon nanotube	Sensitivity 98%	^109^
2.	Ebola	Chronoamperometric	Ebola DNA	RCA‐MB‐Gox‐SPE	–	^110^
3.	Hepatitis B virus	Chronoamperometry	HBe Ag	PdCu TPs/PG	LOD 20 fg ml^−1^	^111^
4.	Hepatitis viruses	Chronoamperometry	Hepatitis B surface antigen (HBsAg) and hepatitis C core antigen (HCVcAg)	GO	LOD 18.2 pg ml^−1^ for HBsAg and 1.19 pg ml^−1^ for HCVcAg	^112^
5.	Hepatitis E virus (HEV)	EIS, DPV	HEV‐LPs	QD@MHS NPs	LOD: 1.2 fg ml^−1^	^113^
6.	Norovirus (NoV)	Photoelectrochemical (PEC)	Recombinant NoV capsid protein VP1	CdS QDs	LOD 4.9 pM	^114^
7.	Norovirus (NoV)	Colorimetric and electrochemical	NoV‐LPs	VONP‐LPs	LOD: 4.1 fg ml^−1^	^115^
8.	Avian Influenza virus	CV	AIV NP	3D nanostructured porous silica film	LOD 1.13 nM	^117^
9.	Avian Influenza virus	EIS	H1N1 and H3N2	Boron‐doped diamond	LOD of 0.33–0.91 pfu	^119^
10.	Human Papillomavirus (HPV)	LAMP/CRISPER/Electrochemical	HPV 18‐activated Cas12a	Au leaf electrodes	1.2 × 10^4^ total copies	^118^
11.	Human Immunodeficiency virus (HIV)	EIS	HIV marker p24 antigen detection	ZnO NWs	LOD: 0.4 pg ml^−1^	^120^
12.	HIV and Coronavirus	Amperometry	H1N1 HA, SARS‐CoV‐2 spike protein	SPCE	LOD for H1N1: 1.12 unit ml^−1^, LOD for SARS‐CoV‐2 S protein 0.15 ng ml^−1^	^121^
13.	Coronavirus	DPV	SARS‐CoV‐2 antigen and antibodies	GO‐Au nanocompiste	LOD (antigen): 3.99 ag ml^−1^ LOD (antibody): 1.0 fg ml^−1^	^122^
14.	Coronavirus	EIS	SARS‐CoV‐2 nucleocapsid protein	MoS_2_‐PDA	2.80 ag ml^−1^	^123^
15.	Coronavirus	DPV	SARS‐CoV‐2 antibody	TiO_2_‐CS bio‐nanocomposites	3.42 ag ml^−1^	^124^
16.	Coronavirus	Chronoamperometry	Viral RNA target SARS‐CoV‐2 S gene	eSIREN	7 copies of RNA target per μl	^125^
17.	Coronavirus	DPV	SARS‐CoV‐2 nucleoprotein	MIP	LOD: 15 fM and LOQ: 50 fM	^126^
18.	Coronavirus	DPV	SARS‐CoV‐2 spike protein	1C, 4C aptamers	–	^116^
	Bacteria					
19.	*Staphylococcus aureus*	EIS	α‐hemolysin	Erythrocyte‐camouflaged	1.9 ng ml^−1^	^127^
20.	Tuberculosis	DPV	16 S rDNA of *M. tuberculosis* H37Ra	Ti_3_C_2_	LOD: 20 CFU ml^−1^	^128^
21.	Tuberculosis	Impedance	*M. tuberculosis* DNA	LDG‐NF	LOD: 1fM	^129^
22.	Escherichia coli (*E. coli*)	CV, EIS	*E. coli* O157	Au NPS	LOD 15 CFU ml^−1^	^130^
23.	*Corynebacterium diphtheria*	amperometry	Diphtheria toxoid	Diethyldithiocarbamate	5 × 10^−6^μg ml^−1^	^131^
24.	*Staphylococcus aureus*	DPV	*S. aureus*	2D‐MOF	6 CFU ml^−1^	^132^
25.	Neonatal septicemia	EIS	Serum amyloid A (SAA)	MWCNTs/MnO2NSs/Co3O4NPs	LOD: 0.01 pM	^133^
	Fungus					
26.	Multi‐resistant fungus	DPV, EIS	*Candida auris*	*Ninhydrin*	LOD: 4.5 pg μL^−1^	^134^
27.	*Aspergillus flavus* (mycelia)	Amperometry	catalase (CAT) and Aflatoxin B1 (AFB1)	palladium nanoparticles/carbonized bacterial cellulose	LOD: 0.434 U ml^−1^	^135^
28.	*Ochratoxin A*	DPV	Ochratoxin A (OTA)	AuNPs‐MoOx	LOD: 3.3 fg ml^−1^	^136^
29.	Mycotoxins	Amperometry	zearalenone (ZEN) and fumonisin B1 (FB1)	rMoS2‐Au	LOD: 5 × 10^−4^ ng ml^−1^	^137^
	Parasite					
30.	Malaria	Amperometric	Plasmodium lactate dehydrogenase (PfLDH)	MP‐dsSPCE	LOD: 200 ng ml^−1^	^138^

## 4 INTEGRATED MICROFLUIDIC‐BASED POCT DEVICES

Microfluidics device becomes an emerging platform that possesses microchannels to provide the handling and precise manipulation of small‐volume fluidic samples ranging from the microliter to nanoliters.^139^, ^140^ In a microfluidic chip, the microchannels are functionalized by the target‐specific capturing component (antibody or antigen or enzyme, etc.), where the separation, mixing, purification, and detection of target analytes are done simultaneously. Hence, they offer rapid detection, high sensitivity, and ultra‐low detection limit. Moreover, they can efficiently detect a single cell in a small volume of the specimen. The microfluidic device shows the excellent potential of the miniaturized and integrated platform for the high‐throughput analysis of bioanalytes on a single device. The integration of the microfluidic device with analytical techniques such as electrochemical, fluorescence, SERS, SPR, etc. to generate a quantitative, semi‐quantitative, or colorimetric signal which can be easily interpreted to determine the presence or absence of target analytes.^141^, ^142^, ^143^, ^144^, ^145^

Microfluidics platform offer numerous advantage such as fully automation process which reduces the time‐consuming manual procedure that enables the rapid detection of analytes. However, the portability, multiplex detection, low sample requirement, and disposable nature efficiently improve the performance of the device which can offer on‐site detection and reduces the overall cost of the device.^146^, ^147^ Nevertheless, the wearable microfluidic system have capable of real‐time sampling and analysis of target pathogens which gains research interest in the biosensor application.

**FIGURE 6:**
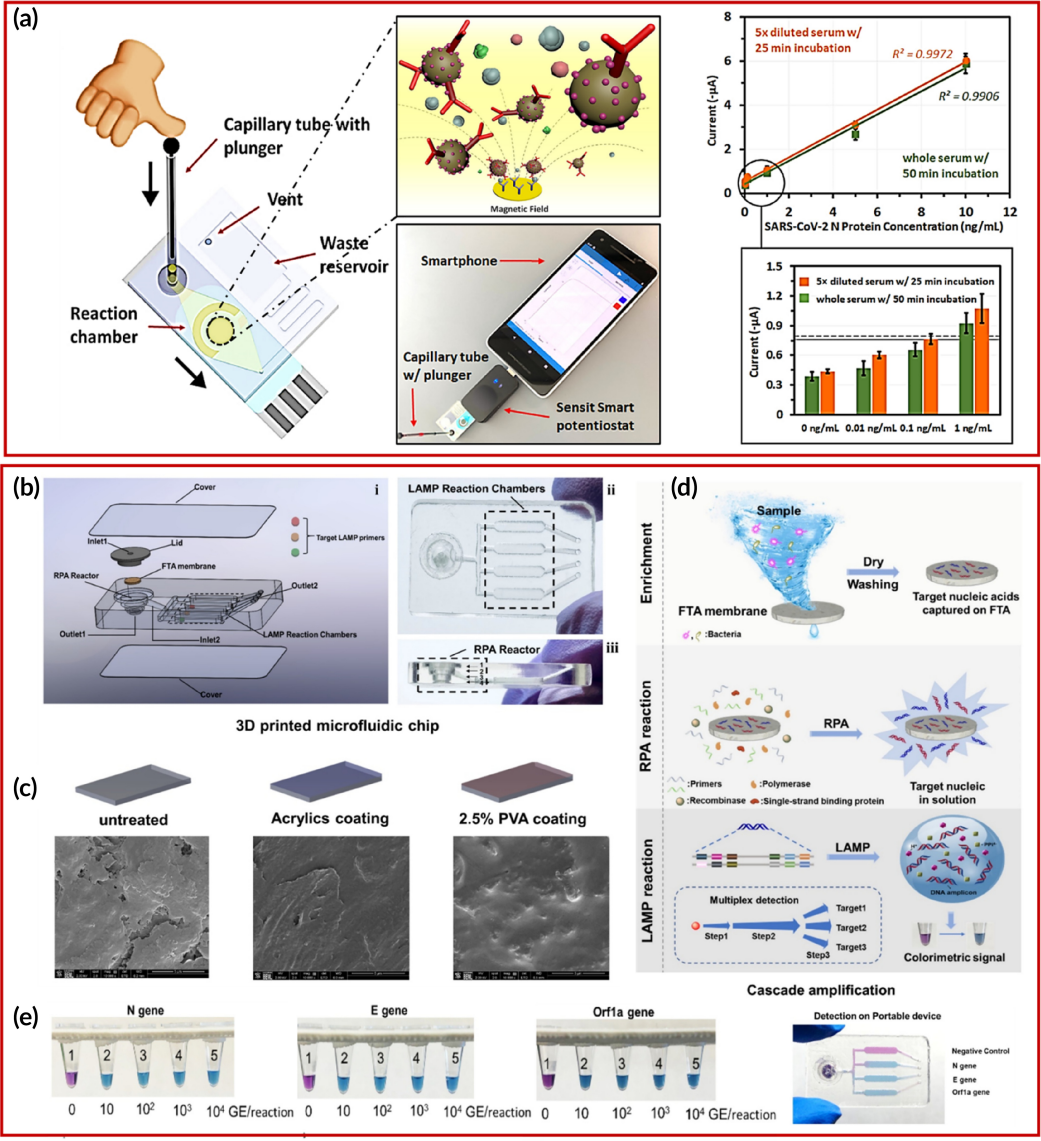
(a) Representation of smartphone‐enabled microfluidic immunosensor chip of magnetic nanoparticles for electrochemical quantification of SARS‐CoV‐2 N‐protein; their calibration graph and amperometric currents response in whole and diluted serum Copyright from Reference 149. (b) An colorimetric‐based microfluidic device for the multiplexed analysis (i: exploded; ii: top; and iii: side view). (c) Surface modification with acrylics and hydrophilic PVA coating and their SEM images. (d) Scheme and optimization of FTA‐based colorimetric kit and (e) Multiplex detection of E, N, and Orf1a of SARS‐CoV‐2 by RT‐RAMP reactions as well as also on a 3D‐printed microfluidic device Copyright from Reference 151.

Magnetic nanoparticles (MNPs) are utilized in biosensing applications since they have high uniformity in shape and size, high surface area, are easy to surface functionalization. Moreover, they have specific binding and capturing of target pathogens in a microfluidic chip. Hence, its outcome in the improvement of the sensitivity and reproducibility of the biosensors. In contrast, MNPs are economic and easily synthesized, therefore, they reduce the overall cost of the device.^148^ For instance, Li et al. proposed a smartphone‐enabled magnetic nanobeads‐based microfluidics device integrated with an electrochemical technique for the detection of SARS‐CoV‐2 N‐protein in serum specimens. Herein, the authors assemble an HRP‐conjugated detecting antibody functionalized magnetic nanobeads as well as an electrode by the functionalization of capture antibody onto the gold electrode. The detection of N‐proteins was done through the amperometric detection technique, where the electro‐catalysis of 3,3′,5,5′‐tetramethylbenzidine (TMB) (TMB conjugated N‐protein) generate an electrochemical signal which quantitatively determines the SARS‐CoV‐2 virus up to pg in the serum sample. The proposed biosensor has high sensitivity and remarkable specificity that can only detect the N‐protein of SARS‐CoV‐2 virus even in the presence of SARS‐CoV‐1 and MERS‐CoV. Furthermore, the integration of the biosensor with a handheld smartphone aid to manage the smart diagnosis of COVID‐19. A detection of SARS‐CoV‐2 N‐protein based on magnetic nanoparticles enables smartphone‐based microfluidic immunosensor chips as illustrated in Figure 6a.^149^

In another report, a paper‐based microfluidics ELISA was developed by Gong and co‐workers for quantitative estimation of IgM/IgG/IgA antibodies in serum. In this paper strip, the detection zone was modified by chitosan and glutaraldehyde coupling agent followed by the immobilization of RBD against the target antibody. The appearance of colors on a paper strip was observed when labeled antibody (anti‐IgM/anti‐IgG/anti‐IgA) captured on RBD protein, which indicates the IgG/IgM/IgA in serum. The colorimetry signal can be interpreted either visually or through a smartphone, so it provides a simple platform to detect the SARS‐CoV‐2 in patients' samples.^150^ Very recently, Yin et al. analyzed the SARS‐CoV‐2 virus in wastewater via colorimetric visualization which was done using a 3D printed microfluidic device integrated with the LAMP technique. This device allows the extraction of nucleic acid on the FTA membrane‐supported chip. Further, the amplification and generation of colorimetric signals simultaneously take place in the reaction zone to quantify the SARS‐CoV‐2 virus. This system provides the multiplex detection platform for E, N, and OrF1a of SARS‐CoV‐2 virus as well as other bacteria. A schematic of the integrated microfluidic chip, their detection process, and a colorimetric view of a reaction mixture on a 3D printed device are shown in Figure 6b–e.^151^

**FIGURE 7:**
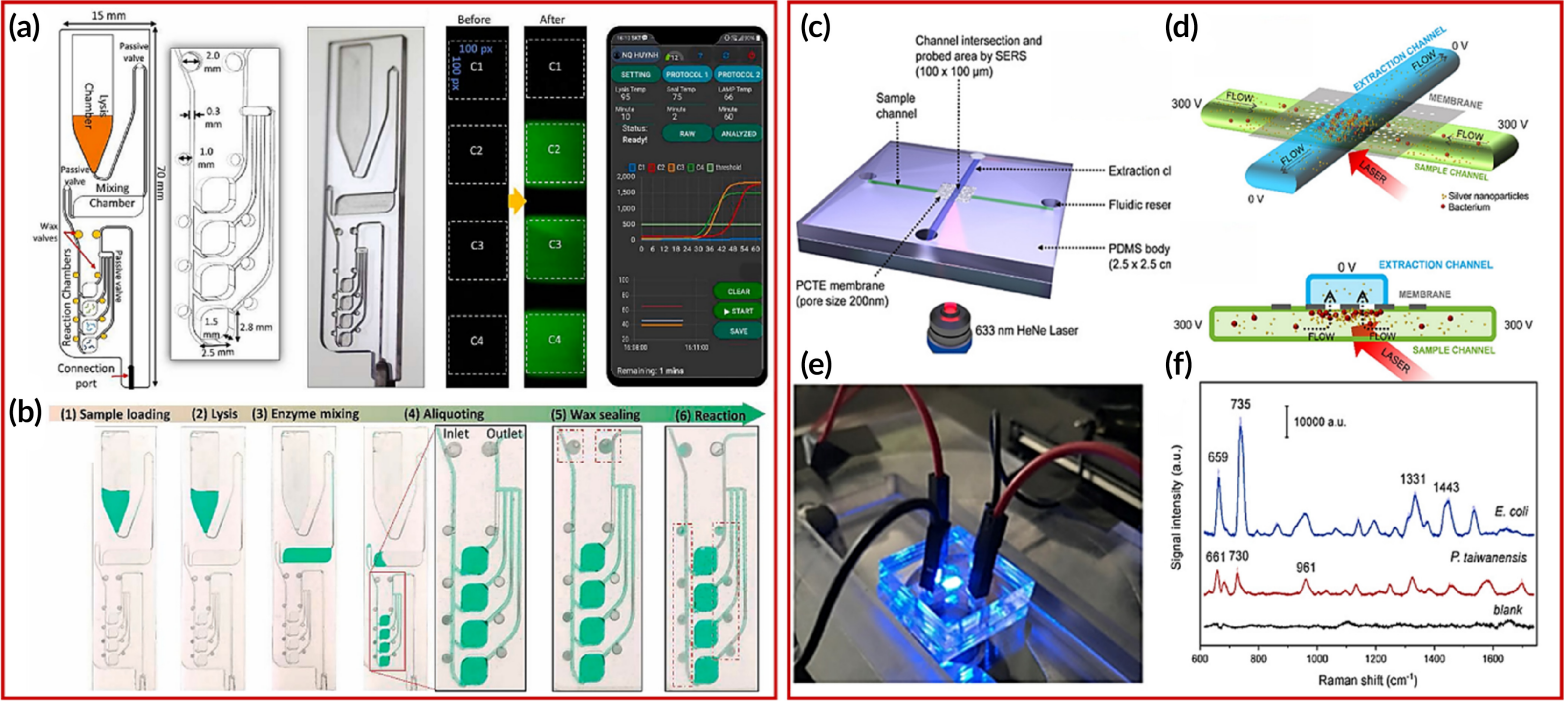
(a) Depiction of the integrated microfluidic device and their digital image; fluorescence images of the reaction chambers before and after the RT‐LAMP reaction (b) Digital images of the operational microfluidic device of a molecular diagnosis on a chip Copyright from Reference 153. The operational procedure of the microfluidic device for (c) the estimation of bacteria followed by SERS detection, (d) its side and cross‐section view, (e) with electrical connections during fluorescence measurement, and (f) SERS spectra of the model bacterial strains Copyright from Reference 167.

Similarly, Yang et al. proposed an RT‐LAMP technique enable a portable test kit similar to a pregnancy kit to sense the RNA of SARS‐CoV‐2 protein. In this detection, the human chorionic gonadotropin (hCG)‐labeled DNA primer was hybridized with the SARS‐CoV‐2 protein and flowed on test‐strip. Where, the positive sample displayed one colorimetric signal on the strip at the control line, since the hCG hybridized SARS‐CoV‐2 protein freely move on the strip which was unable to be captured on the test line. Whereas, the negative control sample displays the two signals on the control as well as test line because of the absence of SARS‐CoV‐2 protein which cannot be hybridized with hCG‐labeled primer and hCG‐labeled primer binds on the test line to appear the signal to indicate the negative sample. This cost‐effective, rapid, and portable test kit can be provided with the onsite detection platform of SARS‐CoV‐2 protein where the test could be even performed by a layman.^152^ Similarly, Nguyen et al. developed the RT‐LAMP integrated disposable microfluidics device assisted with AI for smart monitoring and multiplex detection of SARS‐CoV‐2 virus in swab specimens. The chip was fabricated on a PMMA sheet with the four reaction chambers and then inserted into a handheld portable device followed by the injection of a clinical sample on‐chip. The fluorescent signals are radiated in the presence of three genes As1e, N, and E which indicates the positive result of SARS‐CoV‐2 virus. Additionally, the proposed device is highly specific for SARS‐CoV‐2 virus where it can distinguish even in the presence of other viruses such as RSV A, RSV B, and Influenza A viruses. The schematic of the RT‐LAMP integrated microfluidic chip and its operational processes are shown in Figure 7a, b.^153^

Several reports have shown that a few viruses such as SARS‐CoV‐2 and influenza easily transmitted through the air and could be stable for a long time which infected healthy people. Therefore, the early detection of air‐transmissible pathogens may help to reduce the rate of morbidity.^29^ Xiong et al. reported a centrifugal driving force‐based microfluidics strip for the detection of Orf1ab and N‐gene in aerosol simultaneously; where the appearance of the fluorescent signal indicates the positive sample. They tested the 115 clinical specimens which show excellent specificity of 100%, and sensitivity of 10 copies in the microliter sample.^154^

Very recently, a fast and highly sensitive sandwich assay of the functionalized‐magnetic bead (MBs) and quantum dots (QDs) has been developed for the sensing of enterovirus 71 in a fecal sample. The MBs and QDs were functionalized with the monoclonal antibody against viral envelope protein 1 (VP1) of enterovirus 71. Where the functionalized‐MBs capture the VP1 and then functionalized‐QDs bind to captured VP1 that exhibit the fluorescence emission estimated at the 365 nm UV excitation to evaluate their LOD of 10.0 pg ml^−1^ that has 31 times better than the conventional sandwich ELISA. The very low detection and easy interpretation of results make them more suitable for on‐site detection in clinical samples.^155^ Similarly, Wang et al. reported a magnetic bead functionalized and quantum dots‐based microfluidic chip for multiplex detection of influenza virus and their subtypes (hemagglutinin H7 and H9). This device allows the detection of the influenza virus based on sample separation and estimation of fluorescence intensity when they are conjugated with the anti‐influenza magnetic bead. In a microfluidic device, the larger magnetic bead conjugated with the anti‐influenza subtype is separated firstly in micro‐channels followed by the larger one. After that, streptavidin‐conjugated quantum dots have flowed through the same channel and where they make a sandwich assay. The quantification of fluorescence intensity of quantum dots analyzes the influenza virus and calculated that they have ultra‐low detection ability up to ng ml^−1^ in clinical specimens.^156^

In another context, an electromagnetic integrated digital microfluidics‐based biosensor was employed for the diagnosis of the influenza A (H1N1) virus within 40 min. Herein the microfluidic channels were fabricated by aptamer and antibodies functionalized magnetic bead which generates the fluorescent signal where quantitatively LOD was calculated to be 0.032 HAU in throat swab specimen. Moreover, the fluorescent signal has been enhanced by using tyramide tetramethyl rhodamine which further improves the sensitivity of the biosensor.^157^ Recently, El‐Tholoth et al. proposed a 3D‐printed sensor integrated with the RT‐LAMP for rapid detection of porcine epidemic diarrhea virus (PEDV), transmissible gastroenteritis virus (TGEV), and porcine delta coronavirus (PDCoV) simultaneously in swab specimens of the pig. The developed device has excellent sensitivity, and low detection limit, and allows the detection in a single process. However, further improvements are required to enhance the performance of the assay, since, it usually takes a long time for sample processing and detection as compared with other RT‐LAMP‐based devices.^158^

Similarly, an RT‐LAMP integrated paper‐based microfluidic device has been employed to detect hepatitis C virus. In this work, a conjugate pad of the paper strips was modified by streptavidin‐labeled particles, while the test and control lines were modified by backward loop primer‐labeled fluorescein isothiocyanate, and forward loop primer‐labeled biotin, respectively. The positive sample displays the colorimetric signal on the both test and control line, while the negative sample is only on the control line. The developed sensor is cost‐effective (~$5), rapid, and has been successfully tested in the plasma samples which shows acceptable results.^159^ Similarly, Prabowo et al. proposed a disposable microfluidic test paper where the detection of the dengue virus can be visually observed. Herein, the authors modified the conjugation zone, test, and control line of the paper strip, using an anti‐dengue NS1 antibody, capture antibody, and goat anti‐mouse IgG antibody, respectively. Where the clinical sample containing the dengue virus displays a color spot on the test along with the control line, however, a negative sample can only be at the control line. The advantages of the assay are that it has reliable detection performance in different samples such as serum, cell culture media, and buffer solution.^160^ In another study, a microfluidic device integrated with a fluorometer was proposed for the quick detection of the ebola virus in the blood sample. Herein, clustered regularly interspaced short palindromic (CRISPR) RNA, Cas‐13a, and quenched fluorescent RNA reporter with target ebolavirus RNA were injected into the microfluidic channel.

At the detection zone, the quencher was cleaved by CRISPR‐Cas to generate fluorescent RNA which hybridized with target Ebola virus RNA to generate a fluorescent signal and was measured through the detector within 5 min. The LOD of the assay has been calculated to be 20 PFU ml^−1^ in the blood sample.^161^ Recently, Fu et al. have developed a colorimetric microfluidic chip for simultaneous diagnosis of three biomarkers of swine virus, that is, porcine reproductive and respiratory syndrome virus (PRRSV), classical swine fever (CSV), and porcine circovirus type‐2 (PCV‐2) in four clinical specimens <12 min. This assay has high sensitivity and specificity of 88.8% and 96.6%, respectively, and required 11‐fold less clinical sample for analysis. However, the clinical performance could be further enhanced by the integration of either luminescence or fluorescence techniques instead of colorimetric detection.^162^

Very recently, Song et al. utilized the microfluidics device integrated with the field‐effect transistor (FET) using indium nitride (InN) substrate to detect the HIV antibody in serum up to the picomolar concentration. Authors utilized the rolled‐up technology to make a 3D assembly of the indium nitride through the rolling of a layer of InN on the thin film of silicon which makes the microchannels that have high electron accumulation and enhanced the assay sensitivity. In addition, the tubular channel similarly acts as a capillary channel that flows the sample. However, the InN has a lack of functionalities that affect the binding efficiency and specificity against the target analyte. To overcome this issue, the surface layer of InN was functionalized via the treatment of oxygen plasma followed by a 3‐aminopropyl trimethoxysilane and glutaraldehyde cross‐linker. Then, they were modified by capture antibody to detect the HIV gp41 antibody.^163^

A magnetic microbeads‐based microfluidics platform was projected by Rodoplu et al. for the quantitative detection of *E. coli* bacteria <1 h. First, the *E. coli* bacteria were incubated with the streptavidin‐coated super magnetic beads, where the MBs capture the *E. coli* and then injected into the microfluidic channel which emits the fluorescence images. The utilization of the MBs has several advantages such as they have a high surface‐to‐volume area which captures high loading of bacteria even in the large volume of the samples. Hence, it improved the sensitivity and detection limit. It was found that the proposed assay has excellent detection for *E. coli*, with concentrations ranging from 5 to 5000 CFU ml^−1^. Furthermore, it takes minimum loading and detection time which could serve as an alternative biosensor as compared with the other time‐consuming methods.^164^ Very recently, *Salmonella Typhimurium*, food pathogen bacteria were detected within 1 h on an MBs and metal–organic framework (MOF)‐based fluorescent microfluidic sandwich assay having the LOD of 14.0 CFU ml^−1^. MOF possesses a high surface area, porous nature, and excellent catalytic activity which enhance the sensitivity and detection ability of the assay. The above similar processes were taking place where the target *salmonella Typhimurium* was captured between MBs and NH_2_‐MIL‐101 MOF and they catalyzed the o‐phenylenediamine and hydrogen peroxide to produce yellow color which confirm the presence of *salmonella Typhimurium* in the sample. The authors successfully detected these bacteria in the chicken sample as well with high specificity and a good recovery of rate up to 112%.^165^

Nevertheless, multiplex detection of pathogens in a single device will be a good candidate to avoid the utilization of multiple biosensors and reduce the time consumption and cost for separate detection of analytes. For instance, Chen et al. has been proposed the microfluidic device integrated with the RT‐LAMP for the detection of three bacterial strains of *P. hauseri*, *Salmonella*, and *E. coli* simultaneously. The authors constructed the PDMS microfluidic chip and their four walls are modified by a clone saver card for simultaneous performance of DNA extraction, purification, and sample preparation in a chip. It was found that the assay has excellent sensitivity and a very low LOD of 1.6 cells. Furthermore, the portability and high stability of the assay could beneficial for the on‐site detection of pathogens.^166^ Krafft et al. developed a SERS integrated microfluidic system for simultaneous detection of *E. coli* DH5α and *Pseudomonas taiwanensis* VLB120 in water. The authors did the arrangement of the nanoporous membrane which was perpendicularly connected to the microfluidic channel on the PDMS chip, where both channels work as sample suppliers and sample attraction through potential‐driven force, respectively. However, to further enhanced the surface signal, they utilized the 60–70 nm size of colloidal AgNPs with the targeted sample. The operational principle of the SERS enables microfluidic devices for bacteria enrichment and investigation as shown in Figure 7c–f.^167^

Another infectious disease caused due to fungal infection results in the toxin in food causing multiple health problems like allergy, and organ failure. Asghar et al. reported an integrated microfluidic system modified with the target specific antibody for detection of *Candida albicans* fungus showed linear range of detection from 10 to 10^5^ CFU ml^−1^ with the LOD of 10 CFU ml^−1^ within 2 h. They also examined the high capturing efficacy of the assay for *C. albicans* and it was found that the polyclonal antibody has higher efficacy than a monoclonal antibody. In addition, the low flow rate (5.0 μl min^−1^) has greater capturing efficiency than the high flow rate of 10.0 μl min^−1^. The more capturing of fungus enhances the sensitivity of the assay. The proposed assay is cost‐effective, portable, equipment‐free, and required minimal process to perform the detection as compared with conventional methods.^168^ In another report, Bras et al. estimated the elevated level of three acids such as salicylic acid, azelaic acid, and jasmonic acid against two fungal pathogens, *Botrytis cinerea* and *Erysiphe necator* on a microfluidic device within 7 min in grape and their other products. Since, salicylic acid, azelaic acid, and jasmonic acid are produced in plants against fungal diseases in response to protect them or to fight against the disease. So, the estimation of these acids is the key indicator to knowing about the specific fungal disease. Herein, these three acids were detected using different strategies. Firstly, salicylic acid is detected by the analysis of the shifting of absorbance, when salicylic acid binds to amine‐functionalized‐TiO_2_. Table 2 enlist several recent microfluidic integrated biosensors for the detection of various infection agents.

**TABLE 2:** Microfluidic integrated biosensors for detection of various infectious agents

S. No.	Material	Target	Pathogen	Sample	Technique	LOD/Sens.	Time	References
	Virus							
1.	Carboxylated magnetic bead	N‐protein	SARS‐CoV‐2	Serum	Electrochemical amperometric	50.0 pg ml^−1^	1 h	^149^
2.	Chitosan	IgM/IgG/ IgA	SARS‐CoV‐2	Blood	ELISA	99.7%	‐	^150^
3.	‐	SARS‐CoV‐2, Salmonella typhimurium	SARS‐Co V‐2	Waste water	LAMP	100 GE ml^−1^, 500 CFU ml^−1^	1 h	^151^
4.	HCG‐oligonucleotide probe	RNA	SARS‐CoV‐2	Swab	RT‐LAMP	0.5 copy μL^−1^	‐	^152^
5.	DNA polymerase and primers	As1e, N, E genes	SARS‐CoV‐2	Nasopharyngeal swab	RT‐LAMP	2 × 10^1^ genome copiesμL^−1^	‐	^153^
6.	‐	O and N gene	SARS‐CoV‐2	‐	Fluorescence	10.0 copiesμL^−1^	15 min	^154^
7.	Carboxyl‐functionalized magnetic beads (MBs) and carboxyl‐functionalized quantum dots (QDs)	VP1	Enterovirus 71	Fecal	Fluorescence	10 pg ml^−1^	30 min	^155^
8.	Magnetic bead/quantum dots	Hemagglutinin H7 and H9	Influenza	‐	Fluorescence	3.4 ng ml^−1^ for H7N9, 4.5 ng ml^−1^ for H9N2	‐	^156^
9.	Magnetic bead‐aptamer‐functionalized antibody	H1N1	Influenza A	Throat swab	Fluorescence	0.032 HAU	40 min	^157^
10.	LAMP Primers	‐	PEDV, TGEV and PDCoV	Swab sample of pig	RT‐LAMP	10 genomic copies per reaction for PEDV and PDCoV, and 100 genomic copies per reaction for TGEV	30 min	^158^
11.	LAMP primers	‐	Hepatitis C	Plasma	RT‐LAMP	398 copies/reaction	<40 min	^159^
12.	Poly‐L‐lysine	NS1	Dengue	Serum	Colorimetric	Naked eye, scanner, and a smartphone camera were 200, 46.7, and 74.8 ng ml^−1^	<30 min	^160^
13.	CRISPR‐cas‐13a	RNA	Ebola	Blood	Fluorescence	20 PFU ml^−1^	5 min	^161^
14.	‐	PRRSV, CSFV and PCV2,	Swine	Blood	Colorimetric	88.8% sensitivity	12 min	^162^
15.	Indium nitride	HIV gp41 antibodies	HIV	Serum	FET	2.5 pM		^163^
	Bacteria							
16.	Streptavidin‐modified superparamagnetic microbeads	*E. coli*	Bacteria	‐	Fluorescence	2 CFU ml^−1^	1 h	^164^
17.	(MOF) NH_2_‐MIL‐101(Fe) with mimic peroxidase	Salmonella	Bacteria	Spiked chicken meats	Colorimetric	14 CFU ml^−1^	1 h	^165^
18.	μFAchip	Salmonella, *P. hauseri*	Bacteria	Serum	LAMP	1.6 cells	‐	^166^
19.	AgNPs	*E. coli* DH5α and Pseudomonas taiwanensis VLB120	Bacteria	Drinking‐water	SERS	–	50 s	^167^
	Fungus							
20.	Poly(methyl methacrylate)	*Candida albicans*	Fungus	Blood	Fluorescence	10 CFU ml^−1^	1–2 h	^168^
21.	Salicylic, azelaic and jasmonic acids	Botrytis cinerea and Erysiphe necator	Fungus	Grape plant	Fluorescence	15 μM, 10 μM, and 4.4 nM	7 min	^169^

However, azelaic acid can be detected through the enzymatic reaction catalyzed by the tyrosinase enzyme. Moreover, jasmonic acid was detected via antibody–antigen chemistry. The reported device is very sensitive but needs further modifications to improve its usability to detect the other pathogens in other food products such as fruits, vegetables, etc.^169^ Microfluidic system offers several advantages; besides they struggle with the many challenging factors which further limited their applicability. The major drawback is the analysis of a solid sample that does not dissolve in any solvent may not be detected due to restriction of the flow of sample in micro‐channels. On the other hand, few polymeric materials which react with the microfluidic components may significantly alter the results which change the sensitivity and specificity of the immunosensor chip.

## 5 IOT AND IOMT INTEGRATED ADVANCED SENSORS FOR INFECTIOUS DISEASES

Medical diagnostics, particularly tests for infectious agents such as bacteria, viruses, fungi, and many parasites are well understood and, in fact, constitute an essential component in the medical system to prevent infectious disease spread, thereby reducing public‐health consequences. The development of cost‐effective, easy‐to‐use POC diagnostics to deliver widespread, fast results output independent of laboratories and another medical infrastructure has been the current need of the medical sciences. POC technologies have gained enormous attention and are widely praised among researchers as they have numerous potentials for innovation, such as smartphone‐based platforms which interact with the IoMT more appropriately.

**FIGURE 8:**
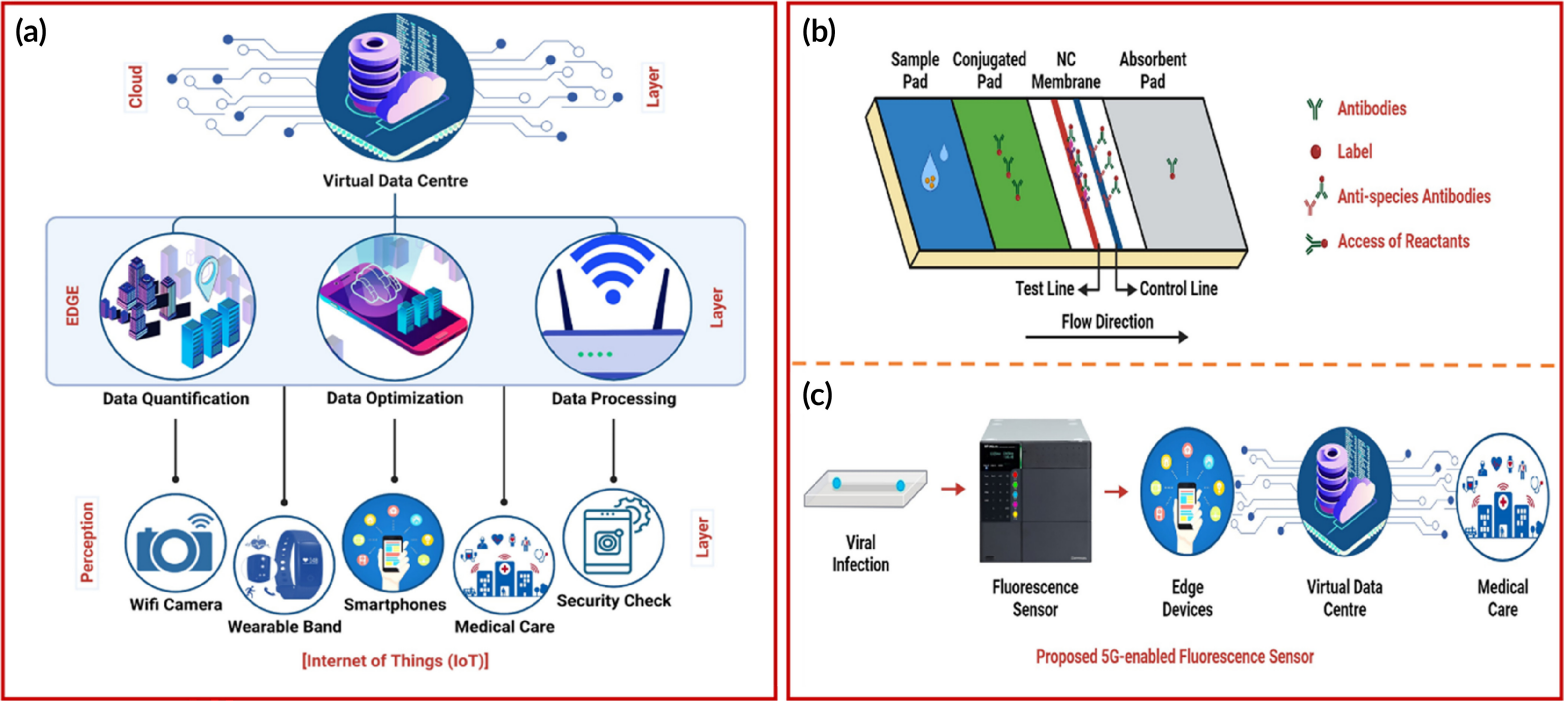
Mechanistic insight on the process of IoMT integrated POCT. (a) Schematic of the 5G‐enabled internet‐of‐medical‐things showing various layers integrated with the virtual data center. (b) The model of lateral flow assay for detection of infectious agents. (c) The working process of the proposed 5G‐enabled fluorescence sensor. IoMT applications of the proposed 5G‐enabled sensor (with a 5G networking circuit module and a data storage circuit module) on a smartphone App for online disease diagnostic and monitoring.

To relieve the burden of diagnosing infectious diseases and encourage intelligent surveillance of suspected patients at remote locations, it is necessary to develop sensitive, selective and smart diagnostic methods for the disease which are caused by the contagious agent responsible for disease community transfer. The IoMT which arose from the IoT, relies on clinical applications of IoT and refers to a podium for connecting medical devices and integrating them into health care networks to circulate relevant appropriate information.^170^ IoMT integrated devices shed light on proactive tele‐healthcare during ongoing COVID‐19 suspects.^171^, ^172^ The growth of IoMT was further enhanced due to the implementation of approaches of ultra‐reliable and low‐latency communications (uRLLC) and massive machine type communications (mMTC).^173^, ^174^, ^175^ In the case of IoMT integrated devices, medical sensors in the perception layer receive and monitor patient data via signals like pressure, light, electricity, humidity, and temperature, then transmit it to the edge and fog layer via ultra‐low latency and high trustworthy wireless communication (Wi‐Fi, cloud data, bluetooth, etc.) before reaching the cloud layer and provide medical institutions and hospitals with the most up‐to‐date medical conditions. Hospitals with limited medical facilities and human resources may benefit from an IoMT‐based smart medical system. The flow chart having different layers in 5G‐enabled internet of medical things' architecture is shown in Figure 8a.

IoMT integrated systems assist patients in receiving quicker and more appropriate medical services. Besides, the integration and handling of large amounts of medical data which is crucial for the prevention and prediction of future pandemics can be taken care of IoMT integrated devices. Furthermore, with the use of IoMT‐based sensors, patients under examination can be kept aware of his/her health state and prompted for regular monitoring. By conducting virtual consultations with specialized doctors, patients can be aware of timely crucial health information.^176^ Recently, an IoMT‐capable and bluetooth‐enabled sensor made it manageable with edge hardware devices. The collected clinical data can be sent with ultra‐low latency and high trustworthiness to the fog layer that is connected to a 5G cloud server. Individuals and hospitals may stay informed about the critical situation of viral infection, which will aid in the treatment and prevention of infectious diseases in the future.

Recently, smart phone integrated sensor which utilizes UCNP@mSiO_2_ as probes for SARS‐CoV‐2 SP and NP quantification showed enhanced sensitivity for SP at a LOD of 1.6 ng ml^−1^ and NP at a LOD of 2.2 ng ml^−1^. The COVID‐19 monitoring component on a smartphone App that works with this sensor allows for online detection, analysis, and monitoring of COVID‐19 patients.^177^ However, well‐established encryption techniques and dependable transmission protocols along with appropriate data transmission and storage facilities to ensure the privacy of clinical subjects and to improve the system's Quality of Service (QoS) is the current demand. As illustrated in Figure 8b, c, the sensor receives the sample solution from the nasopharyngeal swab of the patient.

Virus illness is rapidly spreading from localized outbreaks to national and international distribution. The virus spread among countries has far‐reaching social and economic consequences that go beyond the problems of a single country. To address these issues, several IoT/AI convergence models are being developed. With the advent of convergence technologies such as IT, AI, IoT, and IoMT, researchers are remotely and actively conducting research on disease monitoring, prevention, and containment approach as part of the fourth industrial revolution. Although these studies can be used to prevent disease in a specific region or country, they have limits when it comes to optimizing systems across countries.

The IoT/AI disease monitoring system gathered data from the early phases of infections through the construction of an IoT‐enabled environment, allowing the infection to be dealt with quickly. Furthermore, it assesses the likelihood of transmission and dissemination by comparing the infectious agent's specific disease to existing diseases and gathering respective information through disease surveillance in a specific location. Information is generated when mismatching data with existing microbial infection is recognized, and a new strain prevention control service is supported. An integrated microbial infection control model system can be developed using the IoT/AI convergence disease monitoring system.^91^

The developed model can help identification and monitoring infected individuals or suspect before the spread of infectious disease, as well as analysis of diseases in specific geographical area and countries.^178^ This will enable an early containment and prevention system based on disease outbreaks. The prediction of infectious agent transmission and spread as well as early warning systems for diseases, which provides comprehensive information on pathogen emergence that reflects national and international features could pave the way toward prevention of futuristic pandemics.

## 6 OUTLOOK, CHALLENGES, AND PROSPECTS

The high specificity, ease of mass manufacture, economies, and onsite application, as well as their capacity to offer speedy results, biosensor‐based POC devices have emerged as significant diagnostic approaches for disease diagnostics and therapeutic response monitoring. Traditional approaches are costly, time‐consuming, and labor‐intensive. The biosensors are progressively replacing traditional techniques of analyte detection. For the detection of allergens, toxins, hormones, microbes, pesticides, and other relevant chemicals, they provide a faster, more accurate, and more flexible technique.^179^ The development of the wearable sensor patch devices segment is predicted to increase at the fastest rate. The market for wearable sensors would likely to change dramatically in the future years. The biosensors are predicted to rise in popularity as medical wearable devices become more popular in both medical and consumer markets. Overall, sensing devices, such as lateral flow systems, paper test strips, and electrochemical biosensors, can be utilized for easy screening or selective quantification of materials when combined with computer tools for data gathering and analysis.

The portable readers and information technology integrated instruments such as sensor‐embedded smartphones or portable potentiostat devices with signal transmission capabilities offer the advantage of full quantitative sensing.^180^ For instance, near‐field communications (NFC) for signal readout, is an important advancement in the field of paper‐based sensing tools. These tools allow for data transfer through interaction and integration with the IoT. This provides more stringent communication related to healthcare information in pandemic scenarios, which is important for monitoring, controlling, and halting infection transmission. Moreover, the integration of AI tools with diagnostic systems allowed more fluent management of epidemiological data from a respective area in the COVID‐19 pandemic.^181^, ^182^ The current biosensing scenario appears to be promising.

Every month, new sensor platforms with increased analytical performance are released. Despite this high productivity, bioanalytical gadget development is still largely reliant on trial and error throughout the manufacturing and optimization process. While this technique has shown to be successful thus far, it is important to highlight that the changes are often minor and are only rarely successfully translated to the market. In this regard, we feel that developers may benefit from a more computerized approach that would allow their gadgets to function more analytically efficiently, and effectively.^183^, ^184^, ^185^, ^186^ This will result in a significant reduction in development expenses and a greater likelihood of reaching the product to the market. Aside, using AI models at the time of data analysis may result in a useful tool for improving the sensor's specificity and sensitivity without having to change the biological or hardware components.^183^ Besides the technological hurdles and the lack of a uniform model of disease diagnosis, certain socio‐economic factors need to be considered to establish a large‐scale diagnostic device campaign.

Furthermore, even a high‐sensitivity assay capable of detecting many infectious agent biomarkers, may not be able to produce an unambiguously positive or negative test result in and of itself, because of differences in the patient's specific biomarkers profile which can be linked to many other medical disorders. As a result, a qualified microbiologist should constantly examine the test findings and other symptoms of the patient. In the context of a bulk diagnostic testing survey, this entails the training and deployment of a large number of healthcare professionals, as well as certain modifications to present infrastructures for sample storage and disposal. Another difficulty is balancing the consequences of such a diagnosis on a patient's life. For example, a patient who has been diagnosed with a certain ailment may require psychological assistance, which should be provided as soon as the test results are available. This necessitates the addition of additional professional consultations to follow patients during the whole screening procedure.^187^ Aside from the psychological strain of the examination, the patient must be assured that the results will not be accessible to third parties. Indeed, a data security breach might result in a significant rise in the expense of health care and life insurance, as well as job discrimination. As a result, it is evident that to successfully undertake a large‐scale illness detection program, a strong socioeconomic foundation is required, which can be fulfilled by using additive manufacturing.^188^, ^189^

Chemists and chemical biologists can contribute significantly to the development of biosensing devices against infectious agents which include bacteria, viruses, fungi, and parasites. We still have a lot to learn about the microbial population, their genetics, biochemical pathways, and the mode of interactions with receptors of biological cells. Researchers can use chemical knowledge to link microbial activity to genes and enzymes. At the signaling pathway level, small molecule probes are being produced and utilized to explore microbiota‐immune system interactions. Researchers can use chemical knowledge to link microbial activity to genes and enzymes and hence use it to discover particular microbial components that impact infections or the host immune system.

To fully grasp the potential of biosensing techniques for treating and preventing infectious illnesses, we must solve the significant obstacles of changing the makeup and function of these complex communities. Chemists can assist in the development of next‐generation biosensing strategies via novel material design, which possesses enhanced chemical functionality, high electroconductivity, enhanced surface area, and other tunable physicochemical properties. In addition to their utility as tools, such chemicals may have therapeutic promise. Overall, our increased understanding of the processes underlying the host‐pathogen interaction, along with new technologies for the fabrication of multiplexed, miniaturized, high throughput biosensing tools holds promises to enable innovative strategies to combat infectious disease, a major worldwide health burden.

## 7 CONCLUSION

We must address the major challenges of manipulating the integrated systems and function of these complex devices to fully realize the potential of multiplexed, miniaturized biosensing POC strategies for preventing infectious diseases. Chemists can help develop next‐generation approaches for nano‐material manipulation for the fabrication and development of advanced biosensors, with enhanced sensitivity up to attomolar level and good reproducibility. Such nanomaterials may be the potential for medicinal development in addition to functioning as diagnostic systems. Overall, our growing understanding of the mechanisms behind microbial interaction with human receptors connections, together with emerging tools with the advent of sample manipulation, promises to disclose and enable novel interventions to combat infectious disease, which is a major global health problem. Another unanswered question is how the demand and supply of medical POC diagnostic systems can be fulfilled.

The additive manufacturing‐based 3D bioprinting of POC devices has potential in this regard. It will be crucial to figure out if POCT diagnostics play a role in identifying pathogenic versus nonpathogenic bacteria in the environment. There is still a lot to learn about the process of infection mechanism spread and the role of POCT in germ detection and bacterial defense. We attempted to unify different seemingly unrelated biosensing systems which include electrochemical and microfluidic POCT platforms in this review. The underlying concept, which requires the obstruction of an aperture through which a current is traveling by the analyte species, is the unifying aspect. While the review started with a traditional and commercially available device, it quickly moved on to two fairly recent expressions of the POC sensing paradigm: electrochemical platforms for the detection of viruses, bacteria, fungi, and parasites. The relative benefits and drawbacks of different sensing techniques are worth considering. The capacity to detect an analyte signature via detecting single molecules as they move in and out of the POC system, as well as the ability to use protein engineering to construct a diversity of analyte binding sites in microfluidic channels, are two significant features of the microfluidic integrated approaches.

Furthermore, the advancement in nanomaterial synthetic sciences, on the other hand, has a crucial advantage in the fabrication of mechanically, thermally, and physically stable devices. Additionally, the interior core of the nanomaterial, as well as the chemical environment within the device, can be adjusted at a whim. It seems logical to assume that these two paradigms may be blended to provide a practical sensor that incorporates the best aspects of both. The future of stochastic sensing could be sensor elements that combine the benefits of nanomaterials and BREs into a single entity. Alternatively, functional groups or covalently bonded adapters could be used more efficiently. While these prospects are intriguing, protein engineering's versatility and precision are likely to continue to dominate the field for some time.

Finally, electrical detection does not have to be the only application of stochastic sensing. The principles outlined here could be implemented with the newly available instruments, as single‐molecule detection has advanced dramatically in recent years, particularly by optical and mechanical means. Furthermore, the integration of electrochemical devices on to microfluidic platform coupled with the IoMT platform for big data analysis and cloud computing can be considered a revolution in the field of biosensing. Multiplexed and ultra‐sensitive miniature devices can deliver the needed clinical sensitivity and specificity for infectious disease diagnostics. With the correct ethical and care management, this might reduce the financial load on healthcare systems while also improving microbial infection outcomes. Taken together, advancement in the field of biosensing can emerge as a boon to society and can be useful in combating the present and futuristic epidemics and pandemics in terms of better preparedness.

## AUTHOR CONTRIBUTIONS

**Arpana Parihar:** Conceptualization (equal); formal analysis (equal); project administration (supporting); supervision (supporting); validation (equal); visualization (equal); writing – original draft (equal); writing – review and editing (equal). **Shalu Yadav:** Formal analysis (equal); visualization (equal); writing – original draft (equal); writing – review and editing (equal). **Mohd Abubakar Sadique:** Formal analysis (equal); validation (equal); visualization (equal); writing – original draft (equal); writing – review and editing (equal). **Pushpesh Ranjan:** Formal analysis (equal); validation (equal); visualization (equal); writing – original draft (equal); writing – review and editing (equal). **Neeraj Kumar:** Formal analysis (equal); software (lead); validation (equal); visualization (equal); writing – original draft (equal); writing – review and editing (equal). **Ayushi Singhal:** Formal analysis (equal); writing – original draft (equal); writing – review and editing (equal). **Vedika Khare:** Writing – original draft (equal); writing – review and editing (supporting). **Raju Khan:** Conceptualization (equal); project administration (lead); supervision (lead); validation (equal); visualization (equal); writing – original draft (equal); writing – review and editing (equal). **Sathish Natarajan:** Formal analysis (supporting); validation (supporting); visualization (supporting); writing – review and editing (supporting). **Avanish K. Srivastava:** Validation (supporting); visualization (supporting); writing – review and editing (supporting).

## CONFLICT OF INTEREST

The authors declare no conflict of interest.

### PEER REVIEW

The peer review history for this article is available at https://publons.com/publon/10.1002/btm2.10481.

## Data Availability

Data sharing not applicable to this article as no datasets were generated or analysed during the current study.
